# Bioorthogonal Click of Colloidal Gold Nanoparticles to Antibodies In vivo

**DOI:** 10.1002/chem.202201847

**Published:** 2022-09-01

**Authors:** Christian B. M. Poulie, Emanuel Sporer, Lars Hvass, Jesper T. Jørgensen, Paul J. Kempen, Sara I. Lopes van den Broek, Vladimir Shalgunov, Andreas Kjaer, Andreas I. Jensen, Matthias M. Herth

**Affiliations:** ^1^ Department of Drug Design and Pharmacology Faculty of Health and Medical Sciences University of Copenhagen Jagtvej 160 2100 Copenhagen Denmark; ^2^ Center for Nanomedicine and Theranostics, DTU Health Technology Technical University of Denmark (DTU) Ørsteds Plads 345C 2800 Lyngby Denmark; ^3^ Department of Clinical Physiology Nuclear Medicine & PET, Rigshospitalet Blegdamsvej 9 2100 Copenhagen Denmark; ^4^ Cluster for Molecular Imaging Department of Biomedical Sciences University of Copenhagen Blegdamsvej 3 2200 Copenhagen Denmark; ^5^ National Centre for Nano Fabrication and Characterization Technical University of Denmark (DTU) Ørsteds Plads 347 2800 Lyngby Denmark

**Keywords:** bioorthogonal chemistry, click chemistry, gold nanoparticles, pretargeting, tetrazine ligation

## Abstract

Combining nanotechnology and bioorthogonal chemistry for theranostic strategies offers the possibility to develop next generation nanomedicines. These materials are thought to increase therapeutic outcome and improve current cancer management. Due to their size, nanomedicines target tumors passively. Thus, they can be used for drug delivery purposes. Bioorthogonal chemistry allows for a pretargeting approach. Higher target‐to‐background drug accumulation ratios can be achieved. Pretargeting can also be used to induce internalization processes or trigger controlled drug release. Colloidal gold nanoparticles (AuNPs) have attracted widespread interest as drug delivery vectors within the last decades. Here, we demonstrate for the first time the possibility to successfully ligate AuNPs in vivo to pretargeted monoclonal antibodies. We believe that this possibility will facilitate the development of AuNPs for clinical use and ultimately, improve state‐of‐the‐art patient care.

## Graphical abstract

Combining nanotechnology and bioorthogonal chemistry. Due to their size, nanomedicines target tumors passively. Whereas bioorthogonal chemistry allows for a pretargeting approach. Herein, we demonstrate ‐ for the first time ‐ the possibility to successfully ligate Tz‐AuNPs in vivo to pretargeted monoclonal antibodies.

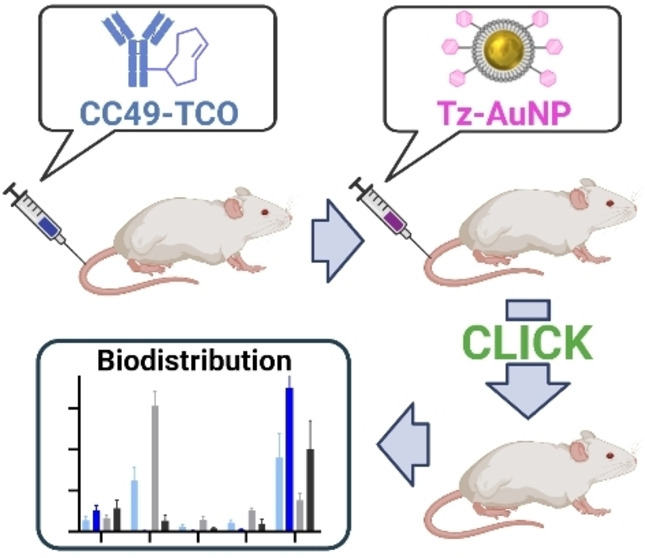

## Introduction

Nanoparticles (NPs) have been explored as advanced tools for biomedical applications.[^1^, ^2^, ^3^] Their unique nanoscale properties enable their use as diagnostics or therapeutics. For example, NPs have been used for photodynamic therapy, magnetic/optical hyperthermia or radionuclide therapy.[^4^, ^5^, ^6^, ^7^] NPs have also been used as contrast imaging agents or for controlled drug release and delivery purposes.[^8^, ^9^] In comparison to conventional systemic drug applications, NPs bear the possibility to protect a drug against degradation, reduce toxicity to healthy tissue and improve target accumulation ‐ among other opportunities. In particular, gold nanoparticles (AuNPs) have attracted widespread interest in the management of cancer.[^7^, ^10^, ^11^] For example, AuNPs have been proposed as theranostic agents, with complementary uses both in therapy and diagnostics, especially within the field of targeted radionuclide therapy.^[12]^ Targeted radionuclide therapy has recently been shown to be effective to treat metastatic castration‐resistant prostate cancer (mCRPC) patients where conventional therapies have failed.[^13^, ^14^] As AuNPs can be stably labeled with the theranostic pair copper‐64/copper‐67[^15^, ^16^] or with other radionuclides for example with the alpha‐emitter astatine‐211,[^17^, ^18^] AuNPs could play an essential role in future theranostic applications. Predicting treatment response is essential for nano‐sized medicines as their tumor accumulation usually depends on the enhanced permeability and retention (EPR) effect an effect based on the passive targeting abilities of nanoparticles into porous tumor tissue, where furthermore the lymphatic drainage is impaired.[^19^, ^20^] Unfortunately, the EPR effect has substantial interpatient and intratumor variability. Tumor uptake can vary for example from 5 % to 50 % ID/kg within the same tumor type.[^21^, ^22^] Consequently, identifying responders from non‐responders is essential for any targeted radionuclide therapy as non‐responders would not benefit from such therapy, but would rather suffer from overall toxicity.[^23^, ^24^] The combination of molecular imaging such as positron emission tomography (PET) or single photon emission computed tomography (SPECT) with targeted radionuclide therapy allows this identification, i. e. to select the right patient before initiating the therapy. The importance of this selection has recently been shown.^[25]^ EPR “positive” patients showed a higher sensitivity to treatment with MM‐302, a liposomal doxorubicin formulation, whereas EPR “negative” patients showed a lower response rate.

**Figure 1:**
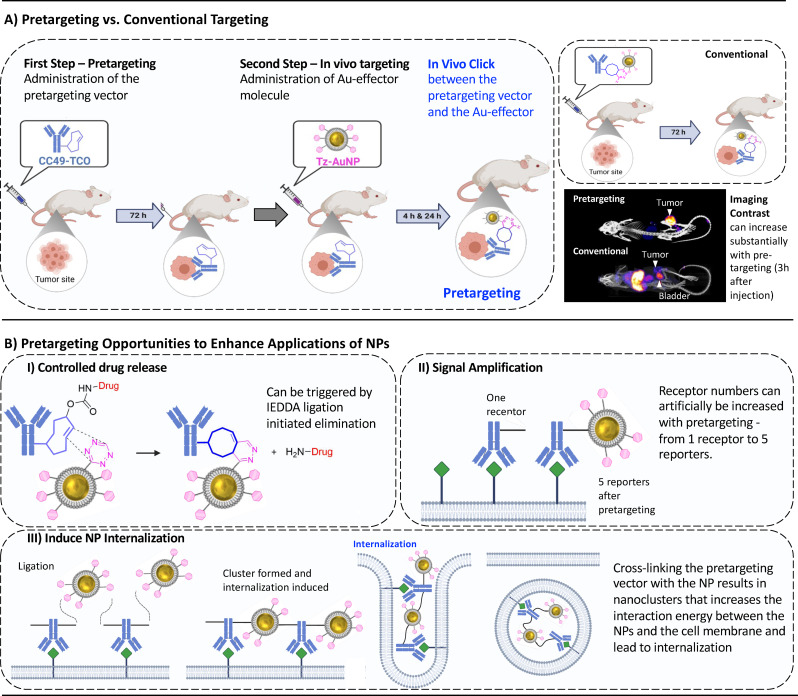
A) The pretargeting concept vs. conventional targeting approaches; Pretargeting is based on a two‐step approach, whereas conventional targeting is based on a single‐step strategy. (This research was originally published in Ref. [34,35], reprinted with permission.[^33^, ^34^, ^35^] B) Pretargeting bears the possibility of enhancing the application of NPs; I) “Click‐to‐release” drug delivery based on pretargeting has recently entered clinical trials (left). The bioorthogonal prodrug activation increased median survival from 26 days to 50 days.[^27^, ^36^] II) NPs can act as signal amplifiers, leading to increased sensitivity of imaging probes or artificial increase the number of biological receptors (right).[^25^, ^26^] For example, pretargeted nanoparticle detection with fluorescence showed a signal of one order of magnitude higher than conventional detection.^[37]^ III) Pretargeting also offers the possibility to induce internalization processes. This can be reached by cross‐linking the pretargeting vector with the NP at the tumor site resulting in nanoclusters that possess an increased interaction energy between the NPs and the cell membrane (bottom).[^38^, ^39^]

Recently, bioorthogonal chemistry has been suggested to overcome certain limitations of nanomedical applications.^[26]^ Bioorthogonal chemistry enables pretargeting.[^23^, ^24^, ^27^] In pretargeting, a targeting vector such as a monoclonal antibody (mAb) is first allowed to accumulate at its target before in a second step a effector molecule is administered that bioorthogonally reacts with the pretargeting vector (Figure 1A).^[28]^ Pretargeting itself has been shown to result in better imaging contrast (target‐to‐background ratio) of up to 125‐fold compared to conventional ImmunoPET,^[26]^ reduce radiation dose to healthy tissue and maximize tolerated dose (Figure 1A).[^23^, ^29^] Pretargeting using mAbs as pretargeting vectors and 6–7 nm organic nanoparticles have already been applied for imaging and targeted radiotherapy purposes.[^30^, ^31^, ^32^]

Pretargeting approaches using NPs with optimal sizes for passive targeting, i. e., in the order of 30–100 nm, have thus far not been realized in vivo.[^40^, ^41^] The combination of pretargeting with the drug delivery capabilities of these nanomedicines (Figure 1B) will improve their therapeutic potential as pretargeting allows for; 1) prodrug activation (Figure 1B, I);[^27^, ^36^, ^42^] 2) increased nanoparticle internalization (Figure 1B, II);^[39]^ 3) signal amplification (Figure 1B, III);[^26^, ^43^] 4) overcoming tumor target heterogeneity;[^26^, ^44^] or 5) in situ drug synthesis.[^27^, ^36^, ^42^] These opportunities especially hold promise for applications in combination therapies,[^45^, ^46^] an approach that has been shown to be more effective compared to monotherapies. For example, “click‐to‐release” selective delivery of doxorubicin given in combination with the immune adjuvant TLR9a increased the median survival of tumor bearing mice to 101 days, compared to 42 days for the respective monotherapies.^[36]^

**Figure 2:**
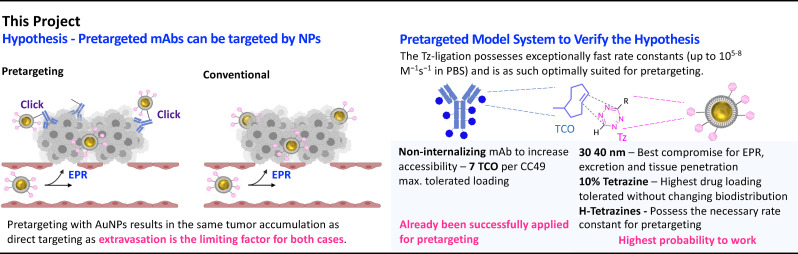
Hypothesis and rationality. Pretargeting and conventional targeting result in the same tumor accumulation as extravasation is the determining factor. Pretargeting is, nevertheless, feasible and leads for example to the beneficial effects displayed in Figure1. In order to verify this hypothesis, we chose a model system with parameters optimal for pretargeting as well as EPR‐based tumor accumulation.

In this project, we investigate the possibility of AuNPs with hydrodynamic diameters of about 30 nm to ligate to pretargeted mAbs in vivo. Recent reports suggest that this might not be possible due to the high internalization capacity of AuNPs.^[47]^ We hypothesized that targeting of pretargeted mAbs with AuNPs is feasible, however, without increasing the absolute tumor uptake as extravasation majorly dependent on the NP size should be the limiting factor in the pretargeted as well as in the conventional targeting case (Figure 2). In order to investigate our hypothesis, we aimed to use PEGylated AuNPs within the size range of 30–40 nm as particles within this size ranged have been reported to show highest tumor uptake within 24 h, relatively fast elimination from the bloodstream and presumably the capacity to penetrate deeper into tumor tissue than NPs>100 nm.[^48^, ^49^] These parameters were considered to be the best compromise between expected absolute tumor uptake due to the EPR effect, fast clearance from the blood to reduce background for potential radionuclide therapy applications and the possibility to reach as many pretargeted mAbs within the tumor.

## Results and Discussion

To enable pretargeting, the tetrazine (Tz) ligation was chosen as it has already been successfully applied for pretargeted strategies.[^33^, ^50^, ^51^, ^52^, ^53^, ^54^, ^55^] Its exceptionally fast second‐order rate constants (up to 10^5–8^ M^−1^s^−1^ in PBS)[^52^, ^55^] allows to reduce the necessary NP concentration to a minimum compared to other bioorthogonal reactions such as the strain‐promoted alkyne‐azide cycloadditions (∼10^−2^ M^−1^s^−1^ in PBS). This leads to a higher probability of the Tz‐ ligation in vivo. Furthermore, “click‐to‐release” drug delivery strategies are feasible making this ligation very attractive.[^23^, ^55^] To maximize the reaction efficiency between the pretargeted mAb and the tetrazine decorated AuNPs (**3**) as well as preserving the protecting effects of the PEG coating with respect to aggregation, phagocytosis, and to ensure enhanced circulation, we aimed for a 10 % surface Tz coating.[^56^, ^57^] This ratio between lipophilic moieties and polar end‐groups on the coating surface has been shown to be acceptable for in vivo applications ‐ at least without impairing circulation massively.^[58]^ A H‐tetrazine structure was chosen as this structure possesses the necessary rate constant for in vivo click chemistries.[^52^, ^55^] As a pretargeting mAb, we decided to use *trans*‐cyclooctene (TCO) modified CC49 (CC49‐TCO). Approximately 7 TCOs per mAb were conjugated to the mAb, as higher TCO loading has been shown to reduce its affinity. This pretargeting vector has already been successfully applied in vivo and proven to be non‐internalizing.^[35]^ Consequently, the possibility of our Tz‐decorated AuNPs (**3**) to react with the mAb should be maximized. Figure 2 summarizes the hypothesis of this study and the rationality of choosing the suggested model system.

**Figure 3:**
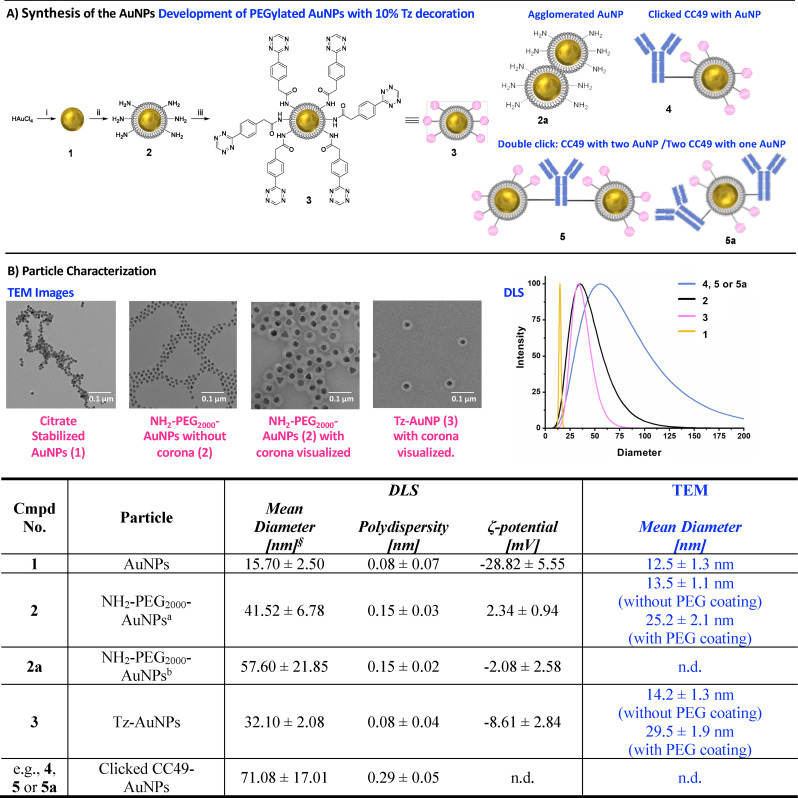
A) Schematic overview of the synthesis approach. Preparation of Tz‐AuNP (left). i) Na_3_Cit ⋅ 2H_2_O, 80 °C, 1 h; ii) HS‐PEG_2000_, HS‐PEG_2000_‐NH_2_, 6 °C, 72 h iii) H−Tz‐NHS, DMSO, H_3_BO_3(aq)_, rt, 4 h, pH 8–9; Observed products beside **3** (right). B) TEM images of the various nanoparticles (upper left); Dynamic light scattering of purified AuNPs (**1**), NH_2_‐PEG_2000_‐AuNPs (**2**), Tz‐AuNP (**3**), Clicked CC49‐AuNPs (**4**) and double‐clicked CC49‐AuNPs (**5**) (upper right); Mean diameter, polydispersity and the *ζ*‐potential is reported and was measured by DLS. Additionally, the mean diameter determined via TEM is reported; n.d.=not determined, [a] AuNPs coating performed at 6 °C, [b] AuNPs coating performed at room temperature, [§] intensity weighted.

Tz‐decorated and PEGylated AuNPs (**3**) were synthesized similar to a procedure published by Llop et al. (Figure 3A).^[47]^ Briefly, AuNPs (**1**) were prepared based on a modified Turkevich‐Frens protocol.[^59^, ^60^] Based on the dynamic light scattering (DLS) and transmission electron microscopy (TEM) derived AuNP size (Figure 3B) and the used starting material, the volume and surface of a single AuNP as well as the total number of AuNPs was calculated. This estimate was used to determine the best ratio between citrate buffer stabilized AuNPs (**1**) and coating material (Supporting Information). 5 equiv. of coating material compared to **1** resulted in the most reproducible coating. With respect to this, a mixture of thiol‐polyethylenglycol‐2000 (HS‐PEG_2000_) and thiol‐polyethylenglycol‐2000‐amine (HS‐PEG_2000_‐NH_2_) (9 : 1) was added to **1** and stirred for 72 h at 6 °C. This ratio was chosen to balance the surface polarity of the AuNP coating, between hydrophilic and more lipophilic end‐groups. Resulting PEGylated AuNPs (**2**) were characterized by DLS and TEM (Figure 3B). Interestingly, PEGylated AuNPs (**2**) coated at room temperature ‐ but using otherwise the same experimental conditions ‐ resulted in 1.4‐fold increased hydrodynamic diameter and in a lower zeta potential, indicating that these AuNPs were agglomerating (Figure 3B). Decoration of **2** with H‐Tzs was carried out by adding 5 equivalents (based on total amount of HS‐PEG_2000_‐NH_2_) of H−Tz‐NHS (**6**, Supporting Information) in aqueous borate buffer at pH 8‐9. The obtained dispersion was stirred for 4 h at ambient temperature. **6** was synthesized in a Pinner‐like synthesis starting from 2‐(4‐cyanophenyl)acetic acid (Supporting Information) followed by conversion of the carboxylic acid into its NHS form.^[53]^ Successful conjugation of the Tz (**6**) to the AuNPs (**2**) was indicated by a drop in zeta‐potential from 2.34±0.94 to −8.61±2.84 (Figure 3B) as the positive surface charge is reduced by conversion of amines to amides. We quantified the amount of accessible Tz via titration with [^64^Cu]Cu‐DOTA‐TCO ([^64^Cu]Cu‐**7**, Supporting Information), which was prepared for this purpose. Approximately 10 % of all PEG chains possessed accessible Tz that could be labeled with [^64^Cu]Cu‐**7**. The ligation yield was not influenced whether the reaction was performed in PBS or mouse serum (Supporting Information). Surface Tz decorated AuNPs could not be prepared directly using pre‐synthesized PEGylated Tz, as the PEGylated Tz appeared to be deactivated by the Au surface and resulted in agglomeration of the AuNPs (Supporting Information). To demonstrate that the Tz‐decorated AuNPs (**3**) synthesized via the two‐step procedure, first PEGylated and then Tz functionalized can react with CC49‐TCO, both structures were mixed in a 1 : 1 ratio in PBS at room temperature and stirred for one hour. A clear increase in the DLS‐derived hydrodynamic diameter was observed (Figure 3B, entry V).^[61]^ Control experiments of Tz‐AuNPs (**3**) with CC49 (without TCOs) did not result in an increase of the AuNPs. These results indicate that **3** reacts quantitatively with CC49‐TCO (Supporting Information).

**Figure 4:**
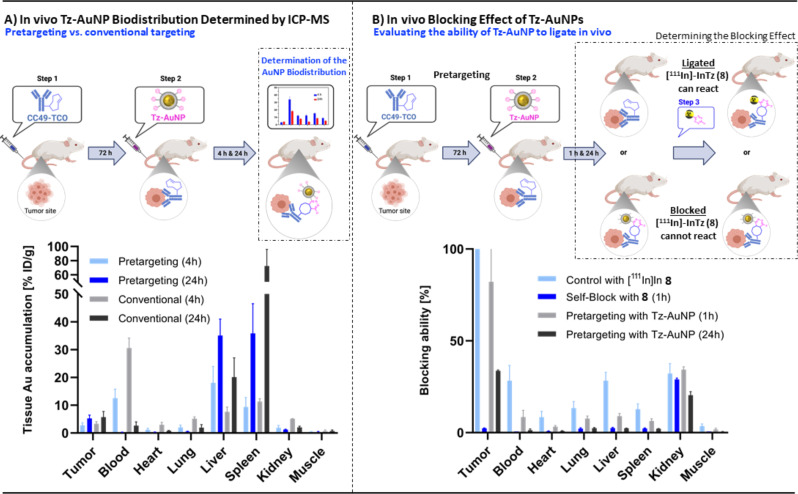
A) In vivo biodistribution, as measured by the Au‐content. Biodistribution was performed 4 and 24 h after administration of Tz‐AuNPs (**3**). Tumor‐bearing mice had been injected with CC49‐TCO, 72 h before administration of the Tz‐AuNPs. B) Blocking study with [^111^In]In‐DOTA‐PEG_11_‐BisPy−Tz ([111In]InTz (**8**)), 1 and 24 h after administration of Tz‐AuNPs (**3**). The blocking effects are normalized to the average tumor uptake of **8**. Tumor‐bearing mice had been injected with CC49‐TCO, 72 h before administration of the Tz‐AuNPs. Biodistribution was performed 22 h post **8** injection.

Encouraged by these results, we investigated the in vivo accumulation of Tz‐AuNPs (**3**) with CC49 or CC49‐TCO pre‐administration. For pretargeting, CC49‐TCO (100 μg/mouse) was administered into LS174T colon carcinoma xenograft bearing BALB/c nude mice 72 h prior to the administration of Tz‐AuNP (**3**) (0.34 μmol, 0.068 mg) (Figure 1A). As control, the same experiment was carried out with CC49 (without TCO modifications). Animals were sacrificed 4 and 24 h post administration of **3** and the AuNP content of the respective tissues were examined. Quantification was carried out by inductively coupled plasma mass spectrometry (ICP‐MS) (Supporting Information). As expected, no difference in the tumor accumulation in the pretargeted and conventional targeting case was observed (Figure 4A). In both cases, approx. 3 % and 5 % ID/g was detected in the tumor tissue after 4 and 24 h, respectively. This indicated that the tumor accumulation is indeed depending on the extravasation of **3** and as such dependent on the NP size and not on the additional active targeting capabilities provided by the pretargeting approach. A significant difference between the pretargeting and the conventional case was observed in blood. At the early time point (4 h), 13 % ID/g was detected in mice preinjected with CC49‐TCO and 31 % ID/g when the unmodified CC49 was preinjected. Interestingly, the reverse trend was observed in the liver, where 18 % ID/g was found in mice preinjected with CC49‐TCO and only 8 % ID/g in the control group. These observations hint on the possibility that Tz‐AuNPs (**3**) are indeed able to ligate to CC49‐TCO, i. e., to the fraction that freely circulates in blood a known observation from other pretargeting experiments with CC49‐TCO.^[58]^ Cross‐linking of Tz‐AuNPs (**3**) to several CC49‐TCOs in blood leads to large nanoclusters, which are prone to be readily cleared by the liver. These formations could explain the observed biodistribution.

In order to investigate if Tz‐AuNPs (**3**) can indeed click in vivo to pretargeted CC49‐TCO, we tested **3**’s in vivo blocking ability to a successfully applied tetrazine imaging agent, namely [^111^In]In‐DOTA‐PEG_11_‐BisPy−Tz **([^111^In]In‐8**, Supporting Information).[^33^, ^51^] The according blocking assay has recently been published by us and allowed to study the relationship between the rate constant and the polarity of the Tz agent with its in vivo performance.^[52]^ The principle behind this assay is based on traditional receptor blocking studies (Figure 4B, Supporting Information). In short, 72 h prior to the injection of Tz‐AuNPs (**3**), CC49‐TCO is administered into BALB/c nude mice bearing LS174T colon carcinoma xenografts. The sequence is completed by administering **[^111^In]In‐8** one and 24 h after Tz‐AuNP (**3**) injection. Determining the blocking effect of Tz‐AuNPs (**3**) on the [^111^In]In‐DOTA‐PEG_11_‐BisPy−Tz (**[^111^In]In‐8**) pretargeting in vivo performance allows to indirectly show the ability of **3** to reach and ligate to CC49‐TCO within the tumor. Tz‐AuNPs (**3**) were able to block tumor uptake of **[^111^In]In‐8** by 18 % one hour after Tz‐AuNP (**3**) administration and 66 % after 24 h (Figure 4B). These data support the ability of Tz‐AuNP (**3**) to ligate with CC49‐TCO in the tumor tissue. The increased blocking ability of Tz‐AuNPs (**3**) over time is in line with the expected extravasation time of the NP into the tumor. Longer timeframes lead to higher accumulation and as such, to a higher ligation degree of **3** to CC49‐TCO. Finally, it must be noted, that the total tumor accumulation increased approximately 50 % in the time course from 1 to 24 h (Supporting Information). This is most likely due to an additional tumor accumulation stemming from in vivo radiolabeled CC49‐TCO in blood at the time point when [^111^In]In‐DOTA‐PEG_11_‐BisPy−Tz (**[^111^In]In‐8**) is injected. This fraction is accumulating via the EPR effect into the tumor over the following 24 h a well‐known behavior at least partly observed for almost all pretargeting approaches.[^50^, ^52^, ^54^]

## Conclusion

In conclusion, we were able to synthesize PEGylated AuNPs functionalized with approximately 10 % Tz (30 nM). These particles were able to ligate to a TCO‐functionalized mAb in vivo. This is to our knowledge the first study that directly shows the capability of NPs>20 nm to ligate in vivo to a mAb within the tumor. This proof‐of‐concept study opens up for novel strategies to improve therapeutic applications of AuNPs from a broader perspective for all kind of NPs to indirectly amplify nanoparticle numbers, overcome tumor target heterogeneity, increase nanoparticle internalization and allow for prodrug activation. We believe that these possibilities will result in NP nanoplatforms with superior efficacy and ultimately, improve current treatment options, particularly for AuNPs.

## Experimental Section

**General information**: All reactions involving dry solvents or sensitive agents were performed under an argon atmosphere and glassware was dried prior to use. Commercially available chemicals were used without further purification. Solvents were dried prior to use with an SG water solvent purification system or dried by standard procedures. Milli‐Q (MQ) water (18.2 MΩ × cm) was used for all AuNP preparation steps. All glassware and magnetic stirring bars were cleaned with freshly prepared aqua regia (HCl: HNO_3_, 3 : 1, v/v) and rinsed five times with MQ water, before use. Reactions were monitored by analytical thin‐layer chromatography (TLC, Merck silica gel 60 F_254_ aluminum sheets). Microwave‐assisted synthesis was carried out in a Biotage Initiator apparatus operating in single mode; the microwave cavity producing controlled irradiation at 2.45 GHz (Biotage AB, Uppsala, Sweden). The reactions were run in sealed vessels. These experiments were performed by employing magnetic stirring and a fixed hold time using variable power to reach (during 1–2 min) and then maintain the desired temperature in the vessel for the programmed time. The temperature was monitored by an IR sensor focused on a point on the reactor vial glass. The IR sensor was calibrated to internal solution reaction temperature by the manufacturer. Automated Flash Column Chromatography was performed on a CombiFlash NextGen 300+ system supplied by TeleDyne ISCO, equipped with RediSep silica packed columns. Detection of the compounds was carried out by means of a UV‐Vis variable wavelength detector operating from 200 to 800 nm and by Evaporative Light Scattering Detector (ELSD). Solvent systems for separation were particular for each compound but consisted of various mixtures of heptane, EtOAc, DCM and MeOH. ^1^H NMR spectra were recorded on a 400 MHz Bruker Avance III or 600 MHz Bruker Avance III HD, and ^13^C NMR spectra on a 101 MHz Bruker Avance III or 151 MHz Bruker Avance III HD. Preparative HPLC was carried out on an Ultimate Thermo SCIENTIFIC HPLC system with an LPG‐3200BX pump, a Rheodyne 9721i injector, a 10 mL loop, an MWD‐3000SD detector (200, 210, 225 and 254 nm), and a Gemini‐NX C18 (250×21.2 mm, 5 μm) column for preparative purifications. Solvent A: H_2_O+0.1 % TFA; Solvent B: MeCN‐H_2_O 9 : 1+0.1 % TFA. For HPLC control, data collection and data handling, Chromeleon software v. 6.80 was used. UPLC‐MS spectra were recorded using an Acquity UPLC H‐Class Waters series solvent delivery system equipped with an autoinjector coupled to an Acquity QDa and TUV detectors installed with an Acquity UPLC®BECH C18 (50 × 2.1 mm, 1.7 μm) column. Solvent A: 5 % aq. MeCN+0.1 % HCO_2_H: Solvent B: MeCN+0.1 % HCO_2_H. Usually, gradients from A : B 1:0 to 1 : 1 (5 min) or A : B 1:0 to 0–50 (5 min), were performed depending on the polarity of the compounds. For data collection and data handling, MassLynx software was used. Compounds were dried under high vacuum or lyophilized using a ScanVac Cool Safe Freeze Drier. For radiolabeling experiments, metal‐free water was used to prepare standard solutions, and metal‐free water was used for all experiments unless otherwise stated. Indium‐111 (III) chloride was purchased from Curium (Netherlands) and copper‐64 (II) chloride was produced at Risø (Denmark). Analytical HPLC was performed on a Dionex system connected to a P680 A pump, a UVD 170 U detector, and a Scansys radiodetector. The system was controlled by Chromeleon 6.8 or Chromeleon 7.2 software. AuNP sizes and Zeta potentials were measured by dynamic light scattering (DLS) on a ZetaPALS (Brookhaven). Metal content of samples was quantified with an ICAP 7000 ICP‐OES (Thermo Scientific). [^64^Cu]CuCl_2_ was produced at the Hevesy Laboratory on a GE PETtrace cyclotron by irradiation of stable ^64^Ni with protons. The irradiated target was dissolved in aq. hydrochloric acid. ^64^Cu was separated from the 64Ni and other impurities by anion exchange chromatography and dried down before use. The [^125^I]NaI was purchased from Perkin Elmer. C57BL6 Mouse serum was purchased from Innovative Research. All glassware and stirbars were cleaned with aqua regia prior to use and rinsed with Milli‐Q water 5 times and dried before use. Radioactivity was quantified with a Princeton Gammatech LGC 5 germanium detector Gamma spectrometer and/or dose calibrator (CRC‐55tR). TLC analysis was performed on MERCK TLC Silica gel 60 F254 aluminum‐backed sheets. Radio‐TLCs were analyzed using a Ray test MiniGita apparatus equipped with a Beta detector GMC, or a Perkin Elmer Cyclone® Plus Storage Phosphor System. Sephacryl 300HR (GE Healthcare) gel filtration medium was used for size‐exclusion chromatography (SEC). AuNPs were concentrated and purified using Amicon Ultra centrifuge filters with a 30 kDa molecular weight cut off (MWCO). Preparation of Boric acid buffer: H_3_BO_3_ (495 mg, 8 mmol) was dissolved in water (50 mL). 0.1 M NaOH (46 mL) was added to adjust the pH to 9.1. Water was added to achieve a final volume of 100 mL and a pH of 9.1–9.3.

**2‐(4‐(1,2,4,5‐Tetrazin‐3‐yl)phenyl)acetic acid (H‐Tz‐COOH)**: 2‐(4‐Cyanophenyl)acetic acid (967 mg, 6.0 mmol, 1.00 equiv.), DCM (385 μL, 6.0 mmol, 1.00 equiv.), sulfur (385 mg, 1.5 mmol, 0.25 equiv.) were dissolved in EtOH (6 mL) and dived over 3 reaction vessels. Hydrazine monohydrate (2.34 mL, 48.0 mmol, 8.00 equiv.) was added slowly under continues stirring. The vessel was sealed and the reaction mixture was heated to 50 °C and stirred for 24 h, behind a blast shield. Then DCM (30 mL) and sodium nitrite (4.14 g, 60.0 mmol, 10.00 equiv.) in H_2_O (60 mL) were added. The two phasic system was cooled to 0 °C and vigorously stirred. Excess AcOH (6 mL) was added dropwise, during which the solution turned bright red. After which, the reaction was allowed to warm to room temperature and was stirred for an additional 30 min. The reaction mixture was extracted with DCM. The organic phase was dried over MgSO_4_. The crude was purified by CombiFlash (MeOH in DCM, 2 % v/v) to afford as a pink solid (360 mg, 1.67 mmol, 28 %). LCMS (ESI) *m/z*=215.1 [M ‐ H]^−^; *R*_f_ =0.19 (95 : 5 DCM/MeOH); ^1^H NMR (400 MHz, MeOD) *δ* 10.31 (s, 1H), 8.54 (d, *J*=8.4 Hz, 2H), 7.57 (d, *J*=8.3 Hz, 2H), 3.76 (s, 2H); ^13^C NMR (101 MHz, MeOD) *δ* 174.7, 167.6, 159.2, 141.6, 132.1, 131.5, 129.2, 41.8.

**2,5‐Dioxopyrrolidin‐1‐yl 2‐(4‐(1,2,4,5‐tetrazin‐3‐yl)phenyl)acetate (H‐Tz‐NHS) (6)**: 2‐(4‐(1,2,4,5‐Tetrazin‐3‐yl)phenyl)acetic acid (100 mg, 0.46 mmol, 1.0 equiv.) was dissolved in dry THF (20 mL). DCC (95 mg, 0.46 mmol, 1.0 equiv.) and *N*‐hydroxysuccinimide (53 mg, 0.46 mmol, 1.0 equiv.) were added to the suspension. The reaction mixture was stirred at room temperature for 20 h. The reaction mixture was filtered, and the clear filtrate was concentrated under reduced pressure. The obtained solid was dissolved in DCM (20 mL) and washed with 2 M Na_2_CO_3_ (2×5 mL). The organic phase was dried over MgSO_4_. The crude was purified by CombiFlash (EtOAc in heptane, 0 to 65 % v/v), which yielded a pink solid (104 mg, 0.33 mmol, 72 %). LCMS (ESI) *m/z*=314.1 [M+H]^+^; *R*_f_=0.27 (1 : 1 EtOAc/heptane); ^1^H NMR (600 MHz, CDCl_3_) *δ* 10.23 (s, 1H), 8.64 (d, *J*=8.4 Hz, 2H), 7.61 (d, *J*=8.6 Hz, 2H), 4.07 (s, 2H), 2.85 (s, 4H); ^13^C NMR (151 MHz, CDCl_3_) *δ* 168.8, 166.2, 166.1, 157.88, 157.86, 136.7, 131.2, 130.4, 128.8, 37.6, 25.6 and is in agreement with previously published data.^[53]^

**(*Z*)‐9‐Oxabicyclo[6.1.0]non‐4‐ene (CCO‐epoxide)**: (*Z*)‐9‐Oxabicyclo[6.1.0]non‐4‐ene was prepared by the following procedure adapted from Clark et al.^[62]^. *cis*,*cis*‐1,5‐Cyclooctadiene (22.0 g, 203.4 mmol, 1.0 equiv.) and dry DCM (300 mL) were added to a 500 mL round‐bottom flask. The mixture was cooled to 0 °C with an icebath and *m*CPBA (45.57 g, 203.4 mmol, 1.0 equiv.) was added step‐wise to give a white suspension. The mixture was heated to room temperature and stirred overnight. The mixture was filtered and washed with sat. NaHCO_3_ (3×100 mL) and sat. NaCl (1×100 mL). The organic layer was collected, dried with MgSO_4_, filtered and concentrated *in vacuo*. Purification by flash chromatography (n‐heptane/EtOAc, 90 : 10) yielded (*Z*)‐9‐Oxabicyclo[6.1.0]non‐4‐ene (11.82 g, 95.2 mmol, 47 %) as a colourless oil. ^1^H NMR (600 MHz, CDCl_3_) *δ* 5.69–5.48 (m, 2H), 3.15–2.91 (m, 2H), 2.55–2.35 (m, 2H), 2.21–2.08 (m, 2H), 2.08–1.86 (m, 4H). ^13^C NMR (151 MHz, CDCl_3_) *δ* 129.00, 56.87, 28.25, 23.82. ^1^H and ^13^C NMR spectroscopic data for were identical to that previously reported.^[62]^

**(*Z*)‐Cyclooct‐4‐enol (CCO‐OH)**: (*Z*)‐Cyclooct‐4‐enol was prepared by the following procedure adapted from Kurra et al.^[63]^ Lithium aluminium hydride tablets (3.26 g, 85.9 mmol, 3.0 equiv.) were added to an oven‐dried 500 mL three‐necked round‐bottom flask. The flask was sealed and flushed with argon. The flask was cooled to 0 °C using an ice‐bath and dry THF (120 mL) was added slowly while vigorously stirring to give a grey suspension. 1,2‐epoxy‐5‐cyclooctene (3.56 g, 28.6 mmol, 1.0 equiv.) in dry THF (10 mL) was added dropwise and the mixture was heated to room temperature and stirred overnight. The mixture was cooled to 0 °C in an ice bath and quenched with EtOAc (120 mL). A sat. solution of Rochelle salt (100 mL) was added, and the mixture was stirred vigorously for 10 minutes. The mixture was transferred to a separatory funnel and the organic layer was collected. The aqueous layer was extracted with DCM (3×150 mL). The combined organic layers were washed with H_2_O (200 mL), dried over MgSO_4_, filtered and concentrated *in vacuo* to give (*Z*)‐Cyclooct‐4‐enol (3.49 g, 28.5 mmol, 99 %) ^1^H NMR (600 MHz, CDCl_3_) *δ* 5.75–5.63 (m, 1H), 5.61–5.52 (m, 1H), 3.86–3.75 (m, 1H), 2.36–2.24 (m, 1H), 2.20–2.04 (m, 3H), 1.97 (s, 1H), 1.93–1.88 (m, 1H), 1.86–1.81 (m, 1H), 1.75–1.68 (m, 1H), 1.67–1.59 (m, 1H), 1.56–1.46 (m, 2H NMR (151 MHz, CDCl_3_) *δ* 130.23, 129.63, 72.85, 37.75, 36.36, 25.75, 24.97, 22.88. ^1^H and ^13^C NMR spectroscopic data were identical to that previously reported.^[62]^

**(*E*)‐Cyclooct‐4‐enol (TCO‐OH)**: (*E*)‐Cyclooct‐4‐enol was prepared by the following procedure adapted from Royzen et al.^[64]^ The bottom of a 220 g column (Flash Cartridge, Screw Top, Luer Lock end fittings, includes top and bottom frit, o‐ring, dispersing insert) was packed with dry silica gel (8 cm), and the top was packed with silver impregnated silica (10 % AgNO_3_, ∼74 g). The column was attached to the pump and the Rayonet® reactor by PTFE tubing and the column was flushed with a diethyl ether/*n*‐heptane mixture (ratio 9 : 1, 500 mL) at a flow rate of 100 mL/min. The column was covered with aluminum to protect the silver from light. The photoreactor and cooler were turned on and the solvent was pumped through the system for 10 minutes. After 10 minutes, the photoreactor was turned off and the system and no solvent and silver leakage was observed. (*Z*)‐Cyclooct‐4‐enol (5.00 g, 39.6 mmol, 1.00 equiv.) was dissolved in a diethyl ether/n‐heptane mixture (ratio 9 : 1, 500 mL) and was added to a 1500 mL quartz flask. Methyl benzoate (5 mL, 25 mmol, 0.06 equiv.) was added to the mixture. The FMI pump was set at a flowrate of 100 mL/min and the Rayonet® reactor equipped with 16×254 nm lamps (RPR‐2537 A) was turned on and photolysis was carried out for 8 h. After 8 h, the photoreactor was turned off and the column was flushed with an additional 200 mL of ether/*n*‐heptane (ratio 9 : 1) and dried with a stream of compressed air. The silica was poured into a 500 mL Erlenmeyer and NH_4_OH (200 mL) and DCM (200 mL) were added. The mixture was stirred vigorously for 10 minutes. The silica was filtered of and the residue was transferred to a separatory funnel. The organic layer was collected, and the aqueous layer was back extracted with DCM (200 mL). The combined organic layers were washed with H_2_O (200 mL), dried over MgSO_4_, filtered and concentrated to give the crude product as a colorless oil (2.432 g). The oil was purified using flash chromatography (n‐heptane/EtOAc 70 : 30) to give the desired compound as separated diastereomers (2.43 g, major isomer 32 % yield, minor isomer 17 % yield). Major product. ^1^H NMR (600 MHz, CDCl_3_) *δ* 5.57 (ddd, *J*=15.3, 11.0, 3.8 Hz, 1H), 5.39 (ddd, *J*=15.5, 11.1, 3.5 Hz, 1H), 3.58–3.37 (m, 1H), 2.36–2.24 (m, 3H), 2.03–1.87 (m, 4H), 1.74–1.45 (m, 3H). ^13^C NMR (151 MHz, CDCl3) *δ* 135.07, 132.79, 77.76, 44.64, 41.10, 34.32, 32.65, 31.22. Minor product. ^1^H NMR (600 MHz, CDCl_3_) *δ* 5.66–5.44 (m, 2H), 4.09–3.94 (m, 1H), 2.47–2.29 (m, 1H), 2.27–2.18 (m, 2H), 2.16–2.03 (m, 2H), 1.91–1.73 (m, 3H), 1.71–1.55 (m, 1H), 1.27–1.20 (m, 1H). ^13^C NMR (151 MHz, CDCl3) *δ* 134.42, 133.17, 67.52, 43.12, 34.23, 34.17, 29.43, 27.80. ^1^H and ^13^C NMR spectroscopic data were identical to that previously reported.^[64]^

**(*E*)‐Cyclooct‐4‐en‐1‐yl (4‐nitrophenyl) carbonate (TCO‐PNB ester)**: (*E*)‐Cyclooct‐4‐enol (104 mg, 0.82 mmol, 1.0 equiv.) was dissolved in DCM (10 mL). The mixture was added to a 10–20 mL microwave vessel, sealed and flushed with argon. Dry triethylamine (0.3 mL, 2.15 mmol, 2.6 equiv.) was added, followed by the addition of *p*‐nitrophenyl chloroformate (209 mg, 1.04 mmol, 1.26 equiv.) in dry DCM (5 mL). The reaction was stirred at room temperature for 30 minutes, after which full conversion was observed by HPLC. Saturated NH_4_Cl (10 mL) was added, and the mixture was transferred to a separatory funnel. The organic layer was separated, and the aqueous layer was extracted with DCM (2×15 mL). The organic layers were combined, dried with MgSO_4_, filtered and concentrated onto Celite. The crude material was purified using CombiFlash purification (12 g silica column, 9 : 1 n‐heptane/EtOAc) to give the expected compound as a white solid (175 mg, 73 % yield). ^1^H NMR (600 MHz, CDCl_3_) *δ* 8.27 (d, 2H), 7.36 (d, 2H), 5.62 (ddd, *J*=15.2, 10.9, 3.8 Hz, 1H), 5.50 (ddd, *J*=16.0, 11.0, 3.5 Hz, 1H), 4.48–4.43 (m, 1H), 2.55–2.34 (m, 3H), 2.25–2.07 (m, 2H), 2.05–1.92 (m, 2H), 1.92–1.84 (m, 1H), 1.81–1.65 (m, 2H). ^13^C NMR (151 MHz, CDCl_3_) *δ* 155.84, 152.11, 145.43, 135.01, 133.19, 125.41, 121.92, 86.58, 40.83, 38.45, 34.25, 32.57, 31.22.

**(*E*)‐Cyclooct‐4‐en‐1‐yl (2‐(2‐(2‐(2‐aminoethoxy)ethoxy)ethoxy)ethyl)carbamate 2,2,2‐trifluoroacetate (TCO‐NH_2_)**: Equatorial (*E*)‐cyclooct‐4‐en‐1‐yl (4‐nitrophenyl) carbonate (TCO‐PNB ester) (25 mg, 0.085 mmol, 1.0 equiv.) was dissolved in dry DMF (100 μL), to which was added 2,2′‐((oxybis(ethane‐2,1‐diyl))bis(oxy))bis(ethan‐1‐amine) (NH_2_‐PEG_3_‐NH_2_) (38 μL, 0.26 mmol, 3.0 equiv.) and Et_3_N (36 μL, 0.26 mmol, 3.0 equiv.). The reaction was stirred at room temperature for 17 h. The reaction vessel was sealed from external light. The reaction was quenched with TFA (0.1 % in H_2_O), filtered and subjected to preparative HPLC. All fractions containing product were combined and lyophilized, which yielded a white solid (34 mg, 0.07 mmol, 86 %). LCMS (ESI) *m/z*=345.2 [M+H]^+^; ^1^H NMR (600 MHz, MeOD) δ 5.60 (ddd, *J*=15.3, 10.4, 4.3 Hz, 1H), 5.47 (ddd, *J*=15.7, 11.1, 3.6 Hz, 1H), 4.35–4.27 (m, 1H), 3.73–3.70 (m, 2H), 3.69–3.66 (m, 4H), 3.66–3.63 (m, 2H), 3.63–3.60 (m, 2H), 3.51 (t, *J*=5.7 Hz, 2H), 3.25 (t, *J*=5.5 Hz, 2H), 3.13 (t, *J*=5.0 Hz, 2H), 2.38–2.27 (m, 3H), 2.03–1.87 (m, 4H), 1.78–1.66 (m, 2H), 1.65–1.53 (m, 1H); ^13^C NMR (151 MHz, MeOD) *δ* 162.4 (q, *J*_C−F_=35.4 Hz), 158.7, 136.1, 133.8, 117.9 (q, *J*_C−F_=291.8 Hz), 81.8, 71.5, 71.4, 71.2, 71.11, 71.08, 67.8, 42.2, 41.5, 40.6, 39.6, 35.1, 33.5, 32.1.

**(*E*)‐2,2′,2′′‐(10‐(1‐(Cyclooct‐4‐en‐1‐yloxy)‐1,15‐dioxo‐5,8,11‐trioxa‐2,14‐diazahexadecan‐16‐yl)‐1,4,7,10‐tetraazacyclododecane‐1,4,7‐triyl)triacetate 2,2,2‐trifluoroacetate (DOTA‐TCO) (7)**: Equatorial (*E*)‐cyclooct‐4‐en‐1‐yl (2‐(2‐(2‐(2‐aminoethoxy)ethoxy)ethoxy)ethyl)carbamate 2,2,2‐trifluoroacetate (30 mg, 0.07 mmol, 1.0 equiv.) was dissolved in dry DMF (100 μL), to which was added Et_3_N (91 μL, 0.65 mmol, 10.0 equiv.) and 1,4,7,10‐tetraazacyclododecane‐1,4,7,10‐tetraacetic acid mono‐*N*‐hydroxysuccinimide ester hexafluorophosphate 2,2,2‐trifluoroacetate salt (DOTA‐mono‐NHS‐ester) (75 mg, 0.10 mmol, 1.5 equiv.). The reaction vessel was sealed from external light and stirred at room temperature for 17 h. Afterwards, the reaction was quenched with TFA (0.1 % in H_2_O), filtered and subjected to preparative HPLC. All fractions containing product were combined and lyophilized, which yielded a white solid (17 mg, 0.02 mmol, 31 %). LCMS (ESI) *m/z*=731.6 [M+H]^+^; ^1^H NMR (600 MHz, MeOD) *δ* 5.60 (ddd, *J*=15.0, 10.3, 4.0 Hz, 1H), 5.47 (ddd, *J*=15.7, 11.1, 3.6 Hz, 1H), 4.38–4.26 (m, 1H), 3.99–3.70 (m, 7H), 3.67–3.59 (m, 9H), 3.57 (t, *J*=5.5 Hz, 2H), 3.51 (t, *J*=5.7 Hz, 2H), 3.44–3.39 (m, 3H), 3.37–3.20 (m, 19H), 2.38–2.28 (m, 3H), 2.02–1.88 (m, 4H), 1.76–1.66 (m, 2H), 1.66–1.55 (m, 1H).

**[^64^Cu]Copper‐(*E*)‐2,2′,2′′‐(10‐(1‐(cyclooct‐4‐en‐1‐yloxy)‐1,15‐dioxo‐5,8,11‐trioxa‐2,14‐diazahexadecan‐16‐yl)‐1,4,7,10‐tetraazacyclododecane‐1,4,7‐triyl)triacetate ([^64^Cu]Cu‐DOTA‐TCO) ([^64^Cu]Cu‐7)**: A stock solution of equatorial (*E*)‐2,2′,2′′‐(10‐(1‐(cyclooct‐4‐en‐1‐yloxy)‐1,15‐dioxo‐5,8,11‐trioxa‐2,14‐diazahexadecan‐16‐yl)‐1,4,7,10‐tetraazacyclododecane‐1,4,7‐triyl)triacetate 2,2,2‐trifluoroacetate (1 mg, 1.4 μmol) in 100 μL DMSO was prepared. 50 MBq of ^64^Cu was dissolved in 100 μL metal‐free NH_4_OAc‐solution (30 mM) and 0.01 M HCl was added to adjust the pH between 5.5–6.8 μL of the stock solution (0.08 mg, 11 nmol) and 92 μL of the ^64^Cu solution were mixed in a metal free vial under vigorous stirring and heated to 50 °C. After 1 h, a TLC indicated the complete chelation of the ^64^Cu. This mixture was used for further experiments without further purification.

**2,2’,2’’‐(10‐(2,40,44‐Trioxo‐44‐((6‐(6‐(pyridin‐2‐yl)‐1,2,4,5‐tetrazin‐3‐yl)pyridin‐3‐yl)amino)‐6,9,12,15,18,21,24,27,30,33,36‐undecaoxa‐3,39‐diazatetratetracontyl)‐1,4,7,10‐tetraazacyclododecane‐1,4,7‐triyl)triacetic acid (DOTA‐PEG_11_‐BisPy‐Tz) (8)**: DOTA‐PEG_11_‐BisPy−Tz was synthesized as previously described by Rossin et al.,^[33]^ starting from 5‐amino‐2‐cyanopyridine and cyanopyridine in six steps. MS (ESI) *m/z*=640.0 [M+2H]^2+^, 426.9 [M+3H]^3+^; ^1^H NMR (600 MHz, MeOD) *δ* 9.08 (d, *J*=2.5 Hz, 1H), 8.89 (dd, *J*=4.8, 0.9 Hz, 1H), 8.80–8.74 (m, 2H), 8.49 (dd, *J*=8.7, 2.5 Hz, 1H), 8.18 (td, *J*=7.8, 1.7 Hz, 1H), 7.74 (ddd, *J*=7.6, 4.8, 1.2 Hz, 1H), 3.88–3.76 (m, 9H), 3.67–3.59 (m, 53H), 3.56 (q, *J*=5.8 Hz, 5H), 3.39 (td, *J*=5.5, 3.0 Hz, 5H), 2.55 (t, *J*=7.3 Hz, 2H), 2.34 (t, *J*=7.3 Hz, 2H), 2.04 (p, *J*=7.4 Hz, 2H); ^13^C NMR (151 MHz, MeOD) *δ* 175.4, 174.3, 164.6, 164.5, 162.2, 151.4, 151.1, 145.2, 142.7, 140.3, 139.6, 128.4, 128.3, 126.2, 125.6, 117.8, 71.56, 71.53, 71.52, 71.50, 71.49, 71.47, 71.45, 71.44, 71.2, 71.07, 70.5, 70.2, 40.3, 36.8, 36.02, 22.5.

**[^111^In]Indium‐2,2’,2’’‐(10‐(2,40,44‐trioxo‐44‐((6‐(6‐(pyridin‐2‐yl)‐1,2,4,5‐tetrazin‐3‐yl)pyridin‐3‐yl)amino)‐6,9,12,15,18,21,24,27,30,33,36‐undecaoxa‐3,39‐diazatetratetracontyl)‐1,4,7,10‐tetraazacyclododecane‐1,4,7‐triyl)triacetic acid ([^111^In]In‐DOTA‐PEG_11_‐BisPy‐Tz) ([^111^In]In‐8)**: Title compound was synthesized as previously described by Edem et al.^[65]^

**AuNPs (1)**: AuNPs (**1**) prepared according to previously published methods, based on a modified Turkevich‐Frens protocol.[^18^, ^59^, ^60^] Tetrachloroauric(III) HAuCl_4_ ⋅ 3H_2_O (100 mg, 253 μmol) was first dissolved in MQ water (50 mL) and added to additional MQ water (650 mL) in a three‐neck flask fitted with a reflux condenser. In parallel, a solution, in a metal‐free plastic tube, of trisodium citrate (588 mg, 2.3 mmol) in MQ water (50 mL) was prepared. The gold solution was then heated to 80 °C, after which the trisodium citrate solution was added in one portion, under vigorous mixing. The reaction mixture was stirred for 1 h at 80 °C, until the color changed to a dark red.

**NH_2_‐PEG_2000_‐AuNP (2)**: Based on the DLS‐derived size, the volume and surface of a single AuNP was calculated. Based on the amount of Au used and the density of Au, the total numbers of AuNPs could be attained. With this total number of AuNPs and the surface area of a single AuNP, the total surface of the AuNPs in the solution was calculated. A total of 4.7 thiol moieties can bind per nm^2^ of AuNP surface.^[66]^ Based on this, the total amount of coating material necessary was calculated. The citrate stabilized AuNPs (**1**) were coated with a 9 : 1 mixture of HS‐PEG_2000_ (9.9 mg, 4.95 μmol) and HS‐PEG_2000_‐NH_2_ (1.1 mg, 0.55 μmol) was weight out, mixed and dissolved in H_2_O (1 mL). Before mixing the citrate stabilized AuNPs (**1**) (6.8 mg, 35 μmol) and PEG‐solutions, both were cooled down to 6 °C. They were mixed at 6 °C, under vigorous stirring and then stored in the fridge for 72 h before use. They were always freshly prepared, and before use, washed with an Amicon® centrifuge filter at 4.4 krpm for 5 min and washed 5 more times.

**Tz‐AuNP (3)**: To NH_2_‐PEG_2000_‐AuNP (**2**) (6.8 mg, 35 μmol) in H_2_O (1.0 mL). Boric acid buffer (0.5 mL) was added to achieve pH 8. 2,5‐Dioxopyrrolidin‐1‐yl 2‐(4‐(1,2,4,5‐tetrazin‐3‐yl)phenyl)acetate (**6**) (1.74 mg, 5.5 μmol, 5 equiv. to the calculated amount of HS‐PEG_2000_‐NH_2_ on the AuNPs) was dissolved in DMSO (10 μL) and added to the AuNPs‐solution and stirred at room temperature for 4 h. After transfer to the centrifuge filter, they were washed 5 times with PBS‐buffer (3.0 mL) until no 2,5‐dioxopyrrolidin‐1‐yl 2‐(4‐(1,2,4,5‐tetrazin‐3‐yl)phenyl)acetate and no 2‐(4‐(1,2,4,5‐tetrazin‐3‐yl)phenyl)acetic acid could be detected on TLC.

**Ex vivo ICP‐MS Measurements of Gold content in Tissue Samples**: Mice bearing LS174T were divided into groups based on their tumor volume (tumor volume ∼100–150 mm^3^, *n*=4 in each group), and administered CC49‐TCO (100 μg/100 μL per mouse ∼7 TCO/mAb, per mouse), or non‐modified CC49, via intravenous tail vein injection. Three days later, mice were intravenously injected with Tz‐AuNPs (**3**). Four or 24 h later, animals were euthanized and tumor, liver, spleen, blood, kidney, lungs, muscle, and heart resected and analyzed. For analysis, between 20–80 mg of each organ were cut into small pieces, and weighted into a 10 mL glass vial. HNO_3_ (500 μL, 16 M), H_2_O_2_ (300 μL, 10 M), and HCl (50 μL, 11 M) were added to each vial, and the vial closed with a metal free lit with a small tube in there to release the overpressure. The samples were stored at room temperature for 48 h until the tissue was completely dissolved. 500 μL was transferred to a metal free 15 mL falcon tube and diluted with 10 mL H_2_O, containing 0.5 ppb iridium, as internal standard. The tumor was further diluted 1 : 10 with 2 % (w/v) HCl solution containing 0.5 ppb iridium. The liver and spleen were diluted 1 : 100 with 2 % (w/v) HCl solution containing 0.5 ppb iridium. Four standards containing 1 ppb, 0.5 ppb, 0.25 ppb and 0.125 ppb gold and 0.5 ppb iridium were prepared. All samples and the standards were measured on the Thermo scientific iCAP Q ICP‐MS and the results expressed as %ID/g (g) calculated via the following equation:

$$\%ID/g=\;\left(\frac{m_{Au}\;in\;tissue}{m_{Au\;}injected}\times 100\;\%\right)/weight\;of\;tissue\;\left(g\right)$$

**Ex vivo blocking assay**: Mice bearing human colon carcinoma xenografts LS174T were divided into groups based on their tumor volume (tumor volumes of ∼100–150 mm^3^, *n*=3–4 in each group) and were administered CC49‐TCO antibodies (100 μg/100 μL, ∼7 TCO/mAb, per mouse). After 3 days, the animals were injected with Tz‐AuNPs (**3**) (Au content: 1.1 mg, 5.2 μmol Tz content:13.2 nmol). One hour or 24 h later, the mice were administered with [^111^In]In‐DOTA‐PEG_11_‐BisPy−Tz ([^111^In]In‐**8**) (∼5 MBq/100 μL, 3.9 nmol) via the tail vein. The mice were euthanized after 22 h and tumor, blood, heart, lung, liver, spleen, kidney, and muscle were resected. All tissues were weighed and the radioactivity measured in a gamma counter (Wizard^2^, Perkin Elmer). Injected amount of radioactivity was corrected for waste and remnants in the syringe after injection, and further corrected to the start of well‐counting. Well‐counting results were decay corrected to the start time of well‐counting, and divided by well‐counter efficiency in relation to the dose calibrator. For calculation of %ID/g, the activity in each tissue sample was divided by tissue weight and then by decay corrected injected activity. Control animals, exclusively receiving [^111^In]In‐**8** were also included. The tumor uptake value from the control group was used for normalization of uptake in other organs, including animals in the other groups.

All animal experiments were performed under a protocol approved by the Animal Research Committee of the Danish Ministry of Environment and Food (license no.: 2016‐15‐0201‐00920) and the Animal Ethics Committee of the University of Copenhagen, and in compliance with the guidelines in Directive 2010/63/EU of the European Parliament on the protection of animals used for scientific purposes.

## Supporting Information

The Supporting Information is available free of charge at XX. Details on the in vitro ligation experiments; Absolute tumor accumulation and TEM images of various AuNPs.

## Authors Contributions

CBMP and ES contributed equally to the work. Tetrazine synthesis was done by CBMP and TCOs were synthesized by CBMP and SILB. AuNP synthesis strategies and subsequent formulation was developed and carried out by ES. TEM was measured by PJK. Radiolabeling was performed by VS. In vivo studies were performed by JTJ and LH. The study was conceptionally designed by CBMP, ES, AK, AIJ and MMH with the help of all the authors. The manuscript was written by CBMP, ES, AK, AIJ and MMH with contribution from all authors. All authors have given approval to the final version of the manuscript.

## Notes

The authors declare no competing financial interest.

## Conflict of interest

The authors declare no conflict of interest.

1

## Supporting information

As a service to our authors and readers, this journal provides supporting information supplied by the authors. Such materials are peer reviewed and may be re‐organized for online delivery, but are not copy‐edited or typeset. Technical support issues arising from supporting information (other than missing files) should be addressed to the authors.

Supporting Information

## Data Availability

The data that support the findings of this study are available from the corresponding author upon reasonable request.
