# Supplementary material for: Bioorthogonal Click of Colloidal Gold Nanoparticles to Antibodies In vivo
Source: Chemistry. 2022 Sep 1;28(61):e202201847. doi: 10.1002/chem.202201847 (PMC9805177; doi:10.1002/chem.202201847)

Chemistry–A European Journal

Supporting Information

Bioorthogonal Click of Colloidal Gold Nanoparticles to Antibodies In vivo

Christian B. M. Poulie, Emanuel Sporer, Lars Hvass, Jesper T. Jørgensen, Paul J. Kempen, Sara I. Lopes van den Broek, Vladimir Shalgunov, Andreas Kjaer,* Andreas I. Jensen,* and Matthias M. Herth*

Contents

Ligation Experiments. 3
RadioTLCs 4
Absolute Tumor Accumulation. 7
TEM Images of Various AuNPs. 8
Images of agglomerated AuNPs. 9
NMR Spectra 10

Ligation Experiments.

Reaction with TCO in PBS. 1 mL of the concentrated Tz-AuNPs (**3**) in PBS (0.7 mg, 3.5 µmol) were mixed with 20 µL of a [^{64}Cu]Cu-DOTA-TCO ([^{64}Cu]Cu-**7**)-solution (0.016 mg, 22 nmol, 2 equiv. to the calculated amount of Tz on the surface) and stirred for 30 min at room temperature (pH 7), after which they were filtered via centrifugation. [^{64}Cu]Cu-**7** was able to ligate with the Tz on the AuNPs the a substantial degree, with 41.2 ± 2.1 % of the [^{64}Cu]Cu remaining on the AuNPs, whereas 57.7 ± 1.9 % of the [^{64}Cu]Cu passed through the filter upon centrifugation as analyzed via TLC, centrifugation filter and PD-10.

Reaction with TCO in mouse serum. 300 µL of the concentrated Tz-AuNPs (**3**) in PBS (0.7 mg, 3.5 µmol) were diluted with further 200 µL of PBS and mixed with 500 µL mouse serum. To this mixture, 20 µL of the [^{64}Cu]Cu-DOTA-TCO ([^{64}Cu]Cu-**7**)-solution (0.016 mg, 22 nmol, 2 equiv. to the calculated amount of Tz on the surface) was added and stirred at room temperature for 30 min. The yield of TCO reacted with the Tz on the surface of the AuNPs was analyzed via radio-TLC.

Reaction with TCO in PBS. 1 mL of the Tz-AuNPs (**3**) (0.07 mg, 0.35µmol) were measured on the DLS and then mixed with CC49-TCO (1.5 µg, 0.01 nmol, 1 mAb per AuNP) and stirred at room temperature for 1 hour and measured again on the DLS. As control, CC49 without TCO was used.

Control experiment show that CC49 and TZ-AuNPs do not react with each other. See table below.

Entry	Particle	Cmpd No.	DLS			TEM
			Mean Diameter [nm]	*Polydispersity [nm]*	*ζ-potential [mV]*	*Mean Diameter [nm]*
**1**	Mixture of CC49 (without TCO) and Tz-AuNPs (**3**)	**CC49 & 3**	39.22 ± 6.95	0.16 ± 0.09	n.d.	n.d.

RadioTLCs

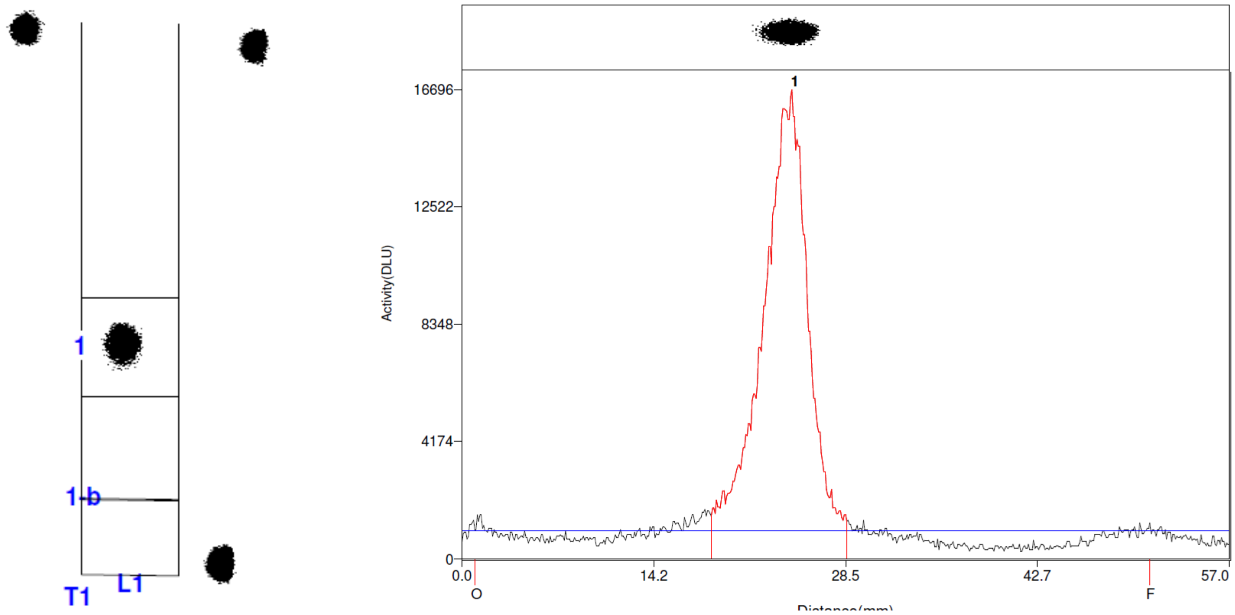

Figure S2. RadioTLC and a representative example of the profile of the synthesis of [^{64}Cu]Cu-DOTA-TCO ([^{64}Cu]Cu-**7**), eluted in 1:1 H_2O : MeOH 5 w/v% NH_4OAc RCP = 100%

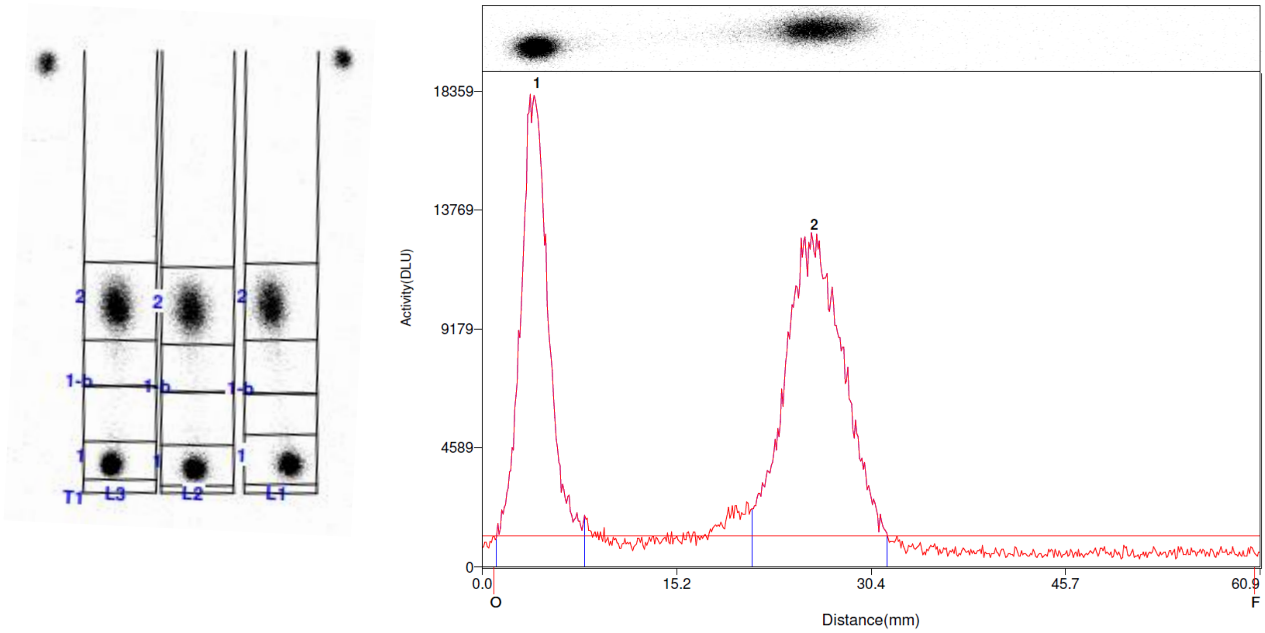

Figure S3. RadioTLCs and a representative example of the profile of the ligation of [^{64}Cu]Cu-**7** with Tz-AuNPs (**3**) in PBS, eluted in 1:1 H_2O : MeOH 5 w/v% NH_4OAc RCC (based on TLC) = 78.3 ± 2.5 %, based on 2 equiv of [^{64}Cu]Cu-**7** and 1 equiv of **3**.

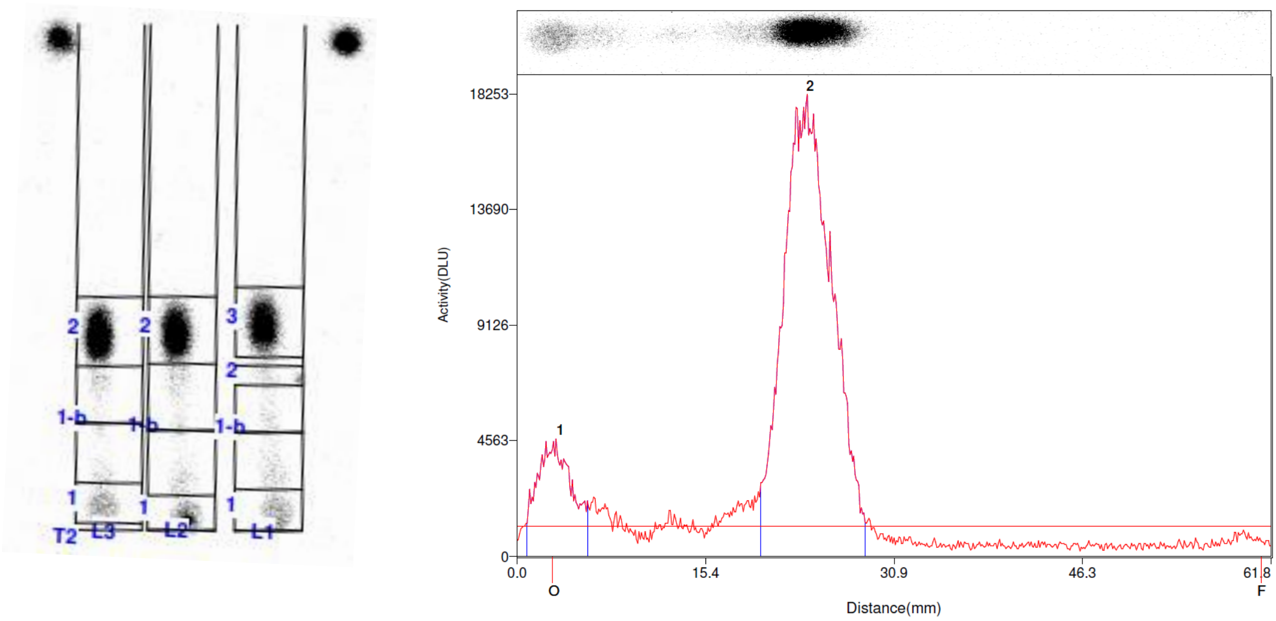

Figure S4. RadioTLCs and a representative example of the profile of the ligation of [^{64}Cu]Cu-**7** with AuNPs (No Tzs) in PBS, eluted in 1:1 H_2O : MeOH 5 w/v% NH_4OAc RCC (based on TLC) = 35.8 ± 5.4 %, based on 2 equiv of [^{64}Cu]Cu-7 and 1 equiv of AuNPs.

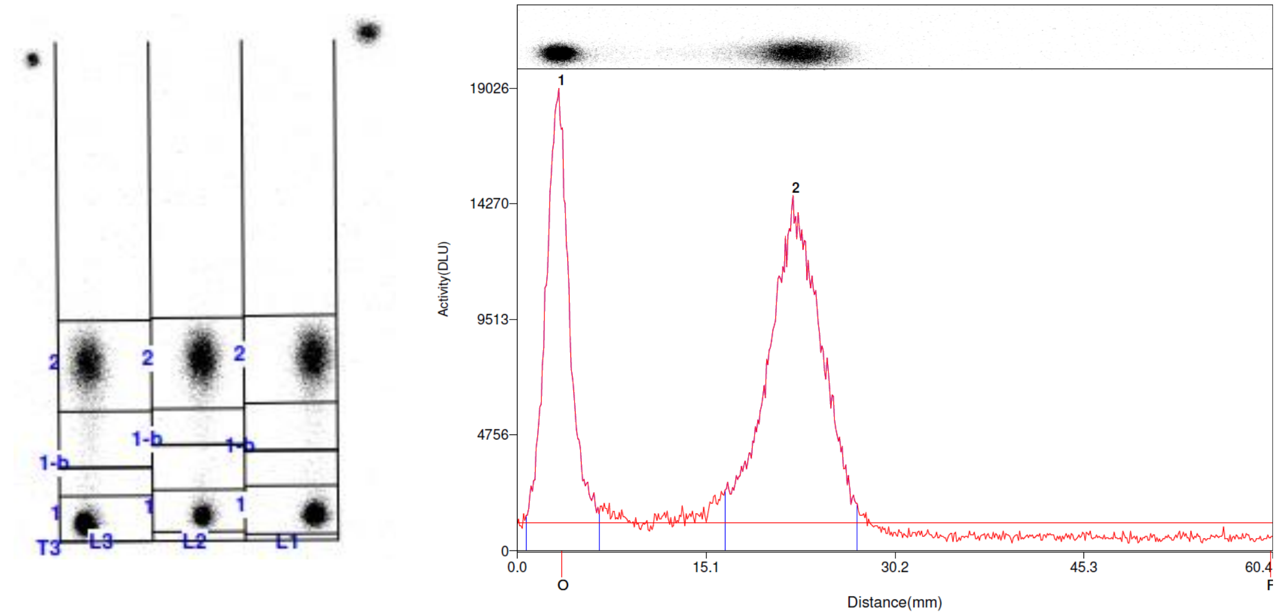

Figure S5. RadioTLCs and a representative example of the profile of the ligation of [^{64}Cu]Cu-**7** with Tz-AuNPs (**3**) in mouse serum, eluted in 1:1 H_2O : MeOH 5 w/v% NH_4OAc. RCC (based on TLC) = 71.4 ± 8.5 %,based on 2 equiv of [^{64}Cu]Cu-**7** and 1 equiv of **3**.

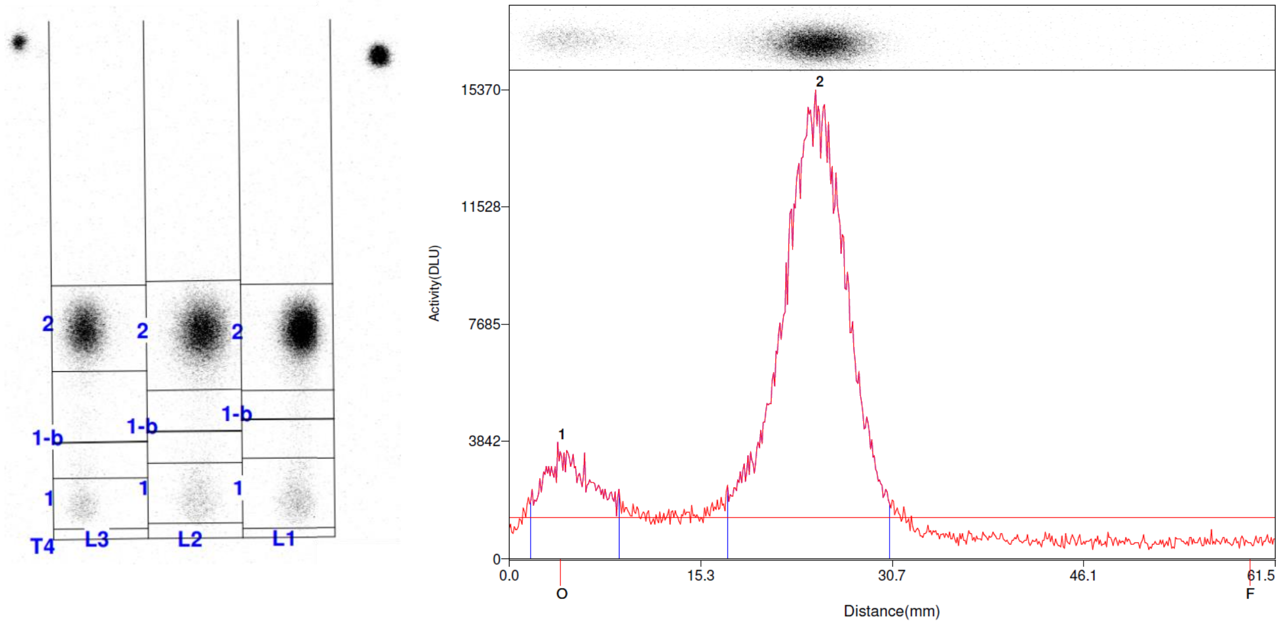

Figure S6. RadioTLCs and a representative example of the profile of the ligation of [^{64}Cu]Cu-**7** with AuNPs (No Tzs) in mouse serum, eluted in 1:1 H_2O : MeOH 5 w/v% NH_4OAc. RCC (based on TLC) = 33.7 ± 1.6 %, based on 2 equiv of [^{64}Cu]Cu-**7** and 1 equiv of AuNPs.

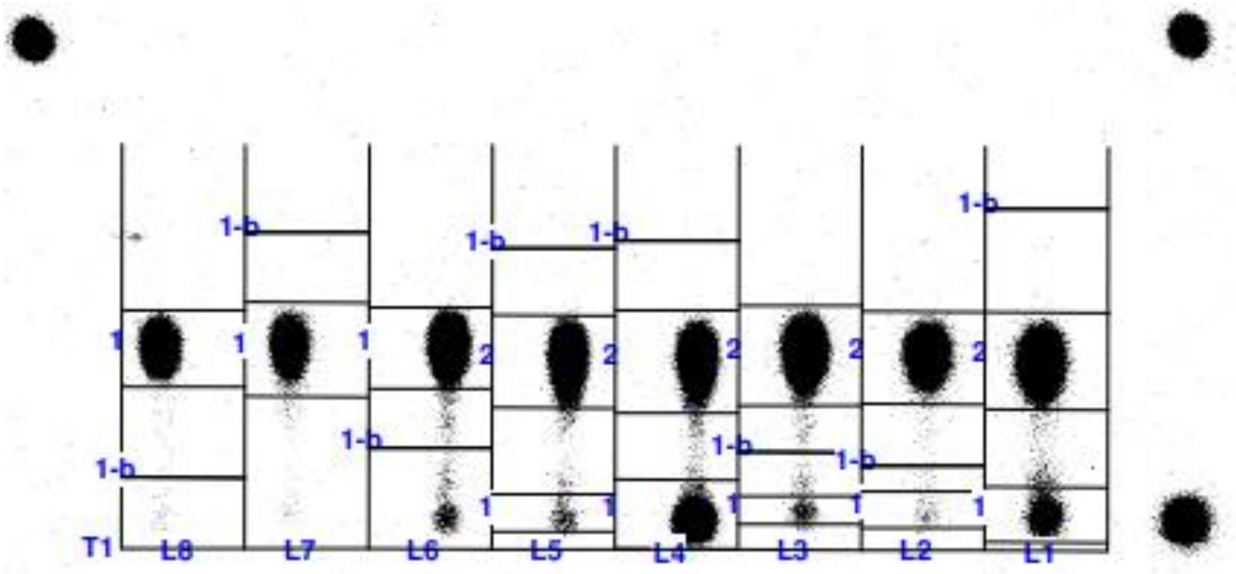

	**L1**	**L2**	**L3**	**L4**	**L5**	**L6**	**L7**	**L8**
AuNPs (starting line) [% sum]	16.7	5.1	4.1	38.1	5.8	3.6	-	-
DOTA-TCO (R_f = 0.4) [% sum]	83.3	94.9	95.9	61.9	94.2	96.4	100	100

Figure S7. RadioTLCs of the optimisation of conjugation of Tz-NHS (**6**) to NH2-PEG$_{2000}$-AuNP (**2**), measured by adding 2 equiv. of [^{64}Cu]Cu-**7**, eluted in 1:1 H_2O : MeOH 5 w/v% NH_4OAc. L1: borate buffer (pH 8.5) / 0% NH_2 (AuNPs coated with PEG$_{2000}$-SH) / Tz-NHS; L2: borate buffer (pH 8.5) / 10% NH_2 (AuNPs coated with 9:1 PEG$_{2000}$-SH : NH_2-PEG$_{2000}$-SH) / Tz-COOH; L3: borate buffer (pH 8.5) / 0% NH_2 (AuNPs coated with PEG$_{2000}$-SH) / Tz-COOH; L4: borate buffer (ph 8.5) / 10% NH_2 (AuNPs coated with 9:1 PEG$_{2000}$-SH : NH_2-PEG$_{2000}$-SH) / Tz-NHS; L5: no borate buffer (pH ~6.5) / 0% NH_2 (AuNPs coated with PEG$_{2000}$-SH) / Tz-NHS; L6: no borate buffer (pH ~6.5)

Absolute Tumor Accumulation.

Table S1. In vivo biodistribution, as measured by the Au-content. Biodistribution was performed 4 and 24 hours after administration of Tz-AuNPs (**3**). Tumor-bearing mice (n=4) had been injected with CC49-TCO, 72 hours before administration of the Tz-AuNPs.

	**Pretargeting (4h)**		**Pretargeting (24h)**		**Conventional (4h)**		**Conventional (24h)**	
	% ID/g	S.D.	% ID/g	S.D.	% ID/g	S.D.	% ID/g	S.D.
Tumor	2.769	0.925	5.265	1.180	3.345	0.748	5.724	1.996
Blood	12.552	3.220	0.234	0.123	30.585	3.574	2.661	1.354
Heart	1.150	0.559	0.300	0.077	2.979	0.864	0.805	0.162
Lung	2.019	0.875	0.470	0.182	5.140	0.671	1.930	1.090
Liver	18.050	5.873	35.089	5.903	7.616	1.745	20.103	6.923
Spleen	9.407	3.409	35.869	10.612	11.310	1.031	72.554	23.298
Kidney	1.899	0.840	1.191	0.182	5.074	0.106	2.067	0.395
Muscle	0.373	0.189	0.355	0.204	0.722	0.392	0.756	0.375

Table S2. Blocking study with [^{111}In]In-DOTA-PEG$_{11}$-BisPy-Tz, one and 24 hours after administration of Tz-AuNPs (**3**). The blocking effects are normalized to the average tumor uptake of [^{111}In]In-DOTA-PEG$_{11}$-BisPy-Tz. Tumor-bearing mice (n=3-4) had been injected with CC49-TCO, 72 hours before administration of the Tz-AuNPs. Biodistribution was performed 22 hours post [^{111}In]In-DOTA-PEG$_{11}$-BisPy-Tz injection.

	**Control with [^{111}In]In-8 (1h) (n=3)**		**Control with [^{111}In]In-8 (24h) (n=3)**		**Self-Block with 8 (1h) (n=3)**		**Pretargeting with Tz-AuNP (1h) (n=3)**		**Pretargeting with Tz-AuNP (24h) (n=4)**	
	% ID/g	S.D.	% ID/g	S.D.	% ID/g	S.D.	% ID/g	S.D.	% ID/g	S.D.
Tumor	3.083	0.908	4.690	0.382	0.073	0.006	2.533	0.961	1.580	0.022
Blood	0.873	0.256	0.840	0.085	0.020	0.000	0.263	0.111	0.065	0.031
Heart	0.260	0.098	0.220	0.028	0.023	0.006	0.100	0.020	0.043	0.010
Lung	0.413	0.110	0.390	0.014	0.067	0.015	0.237	0.042	0.115	0.017
Liver	0.873	0.142	0.815	0.191	0.077	0.012	0.277	0.046	0.110	0.008
Spleen	0.393	0.091	0.440	0.085	0.070	0.01	0.197	0.038	0.100	0.008
Kidney	0.993	0.167	1.060	0.099	0.893	0.023	1.060	0.044	0.958	0.091
Muscle	0.110	0.036	0.100	0.000	0.020	0.000	0.053	0.021	0.025	0.006

TEM Images of Various AuNPs.

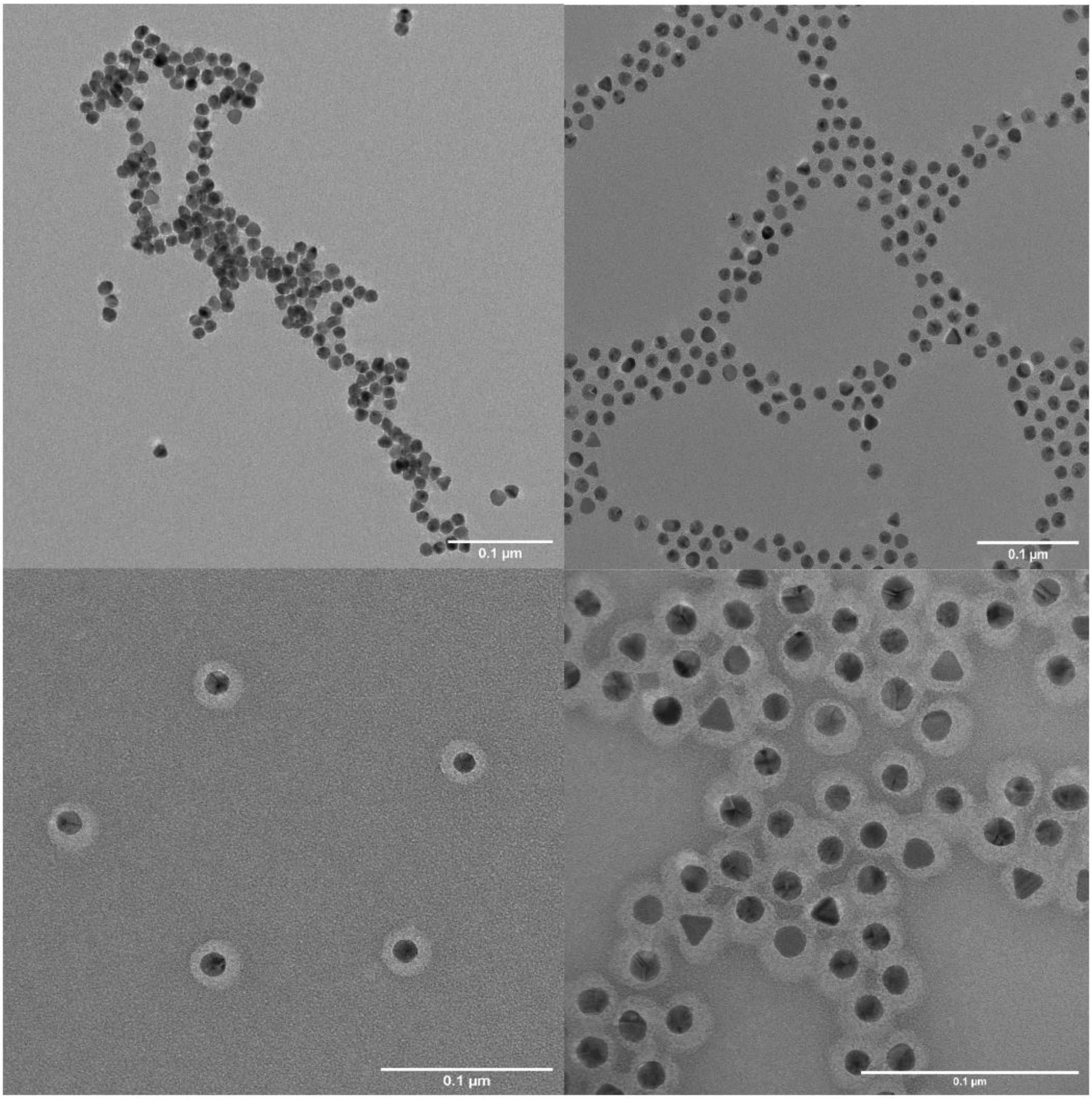

Figure S8: (left upper): citrate stabilized AuNPs (12.5 ± 1,3 nm) (lower left): Tz-AuNPs (without corona 14.2 ± 1.3 nm; with corona 29.5 ± 1.9 nm) (right upper): NH_2-AuNPs (13.5 ± 1.1 nm) (right lower): corona of NH_2-AuNPs visualized. Sample preparation: 3 µL of NP solution was drop cast onto a freshly glow discharged 200 mesh nickel TEM grids coated with carbon stabilized formvar and allowed to adsorb for 5 min before the excess solution was removed via blotting. The grid was then rinsed with distilled water 3 times and placed sequentially on 3 drops of 2% uranyl acetate stain for 30, 10, and 60 seconds before the excess was wicked away with filter paper to negatively stain the sample. The grid was dried and imaged using a Tecnai T20 G2 located at the National Centre for Fabrication and Characterization at Technical University of Denmark. Images were acquired using a TVIPS-XF416 CMOS camera (TVIPS GmbH, Gauting, DE).

Images of agglomerated AuNPs.

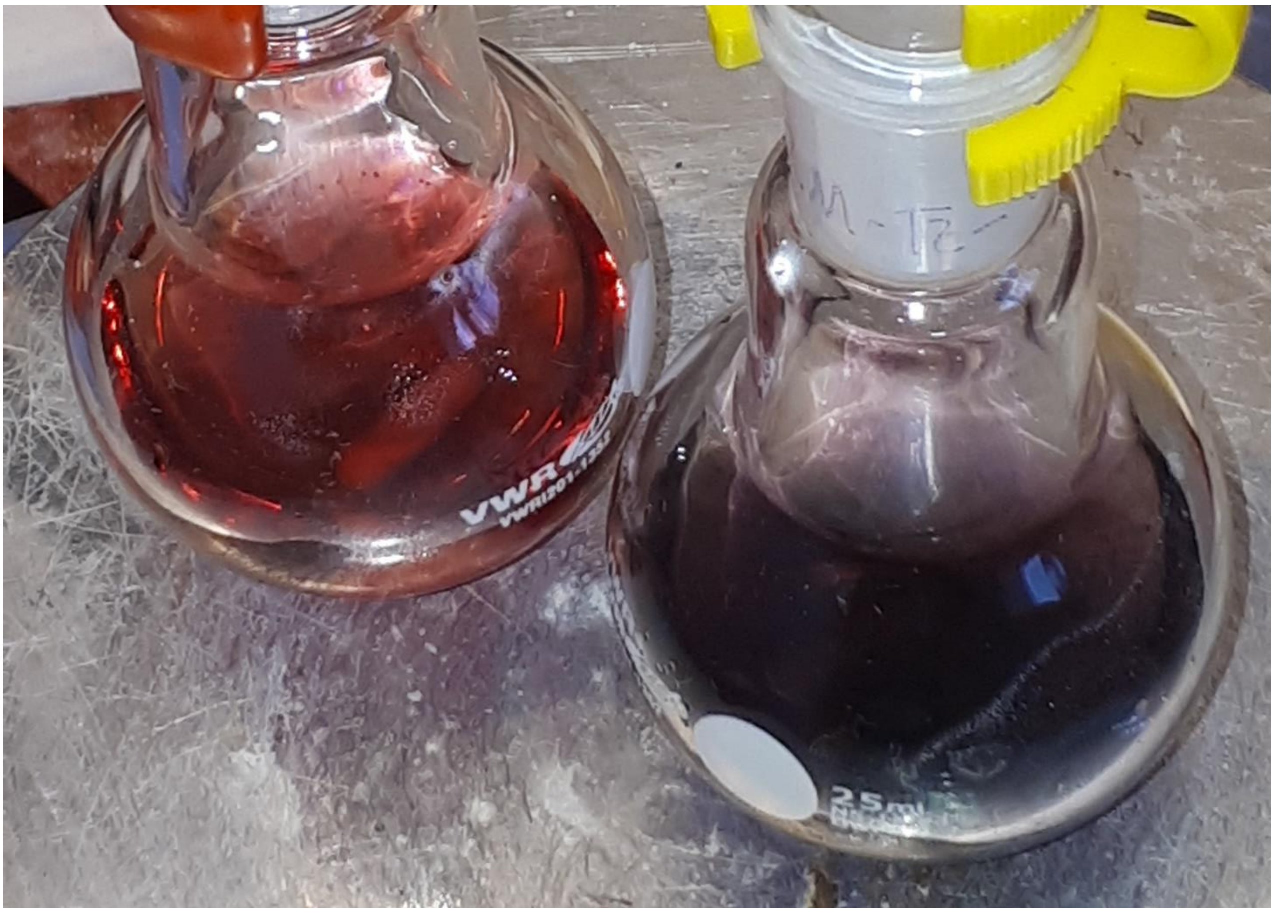

Figure S9: (left): citrate stabilized AuNPs (right): PEGylated Tz added to citrate stabilized AuNPs, clear agglomeration was observed.

NMR Spectra
H-Tz-NHS (6)

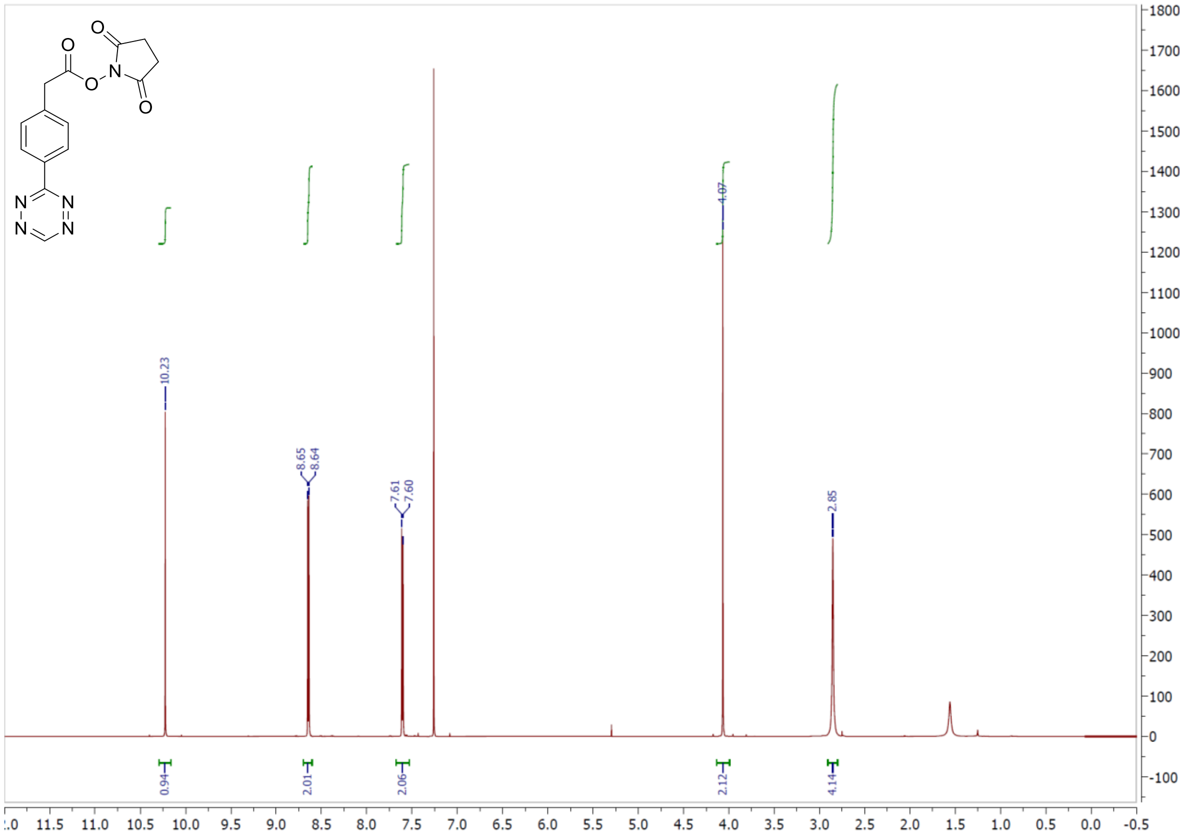

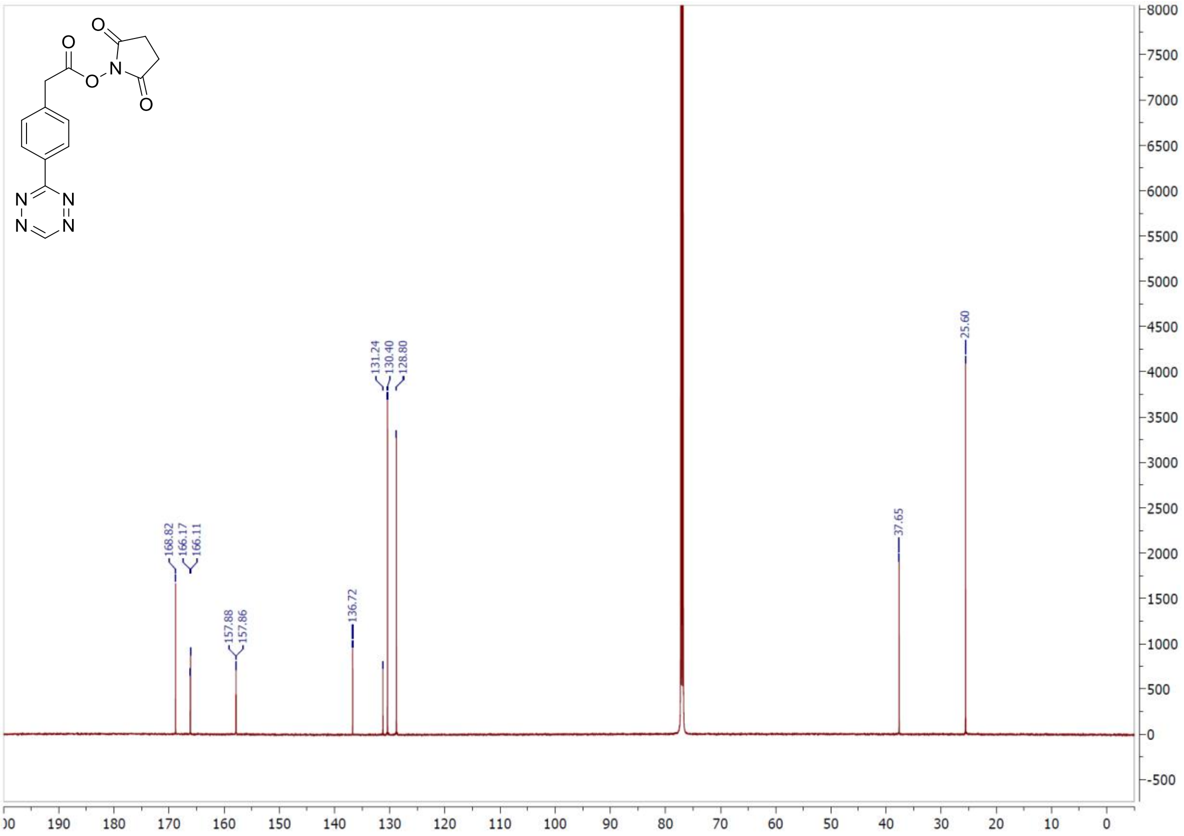

TCO-NH$_2$

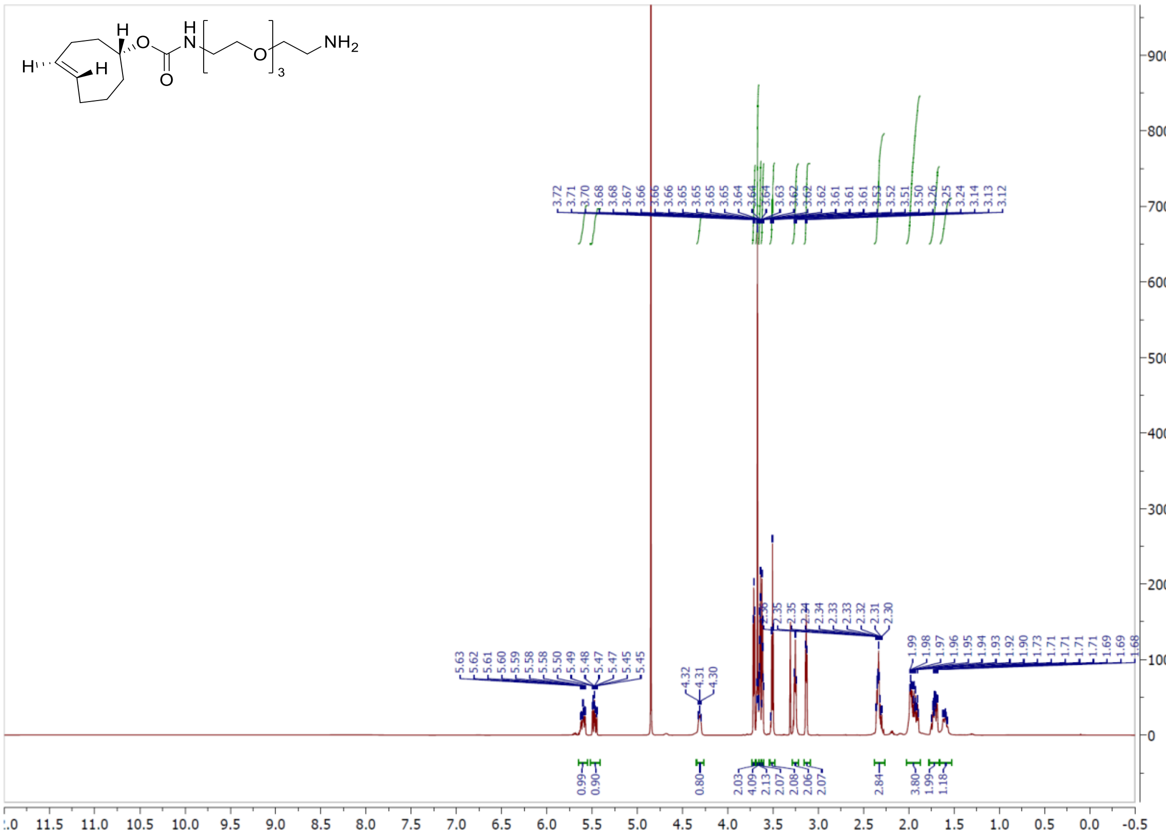

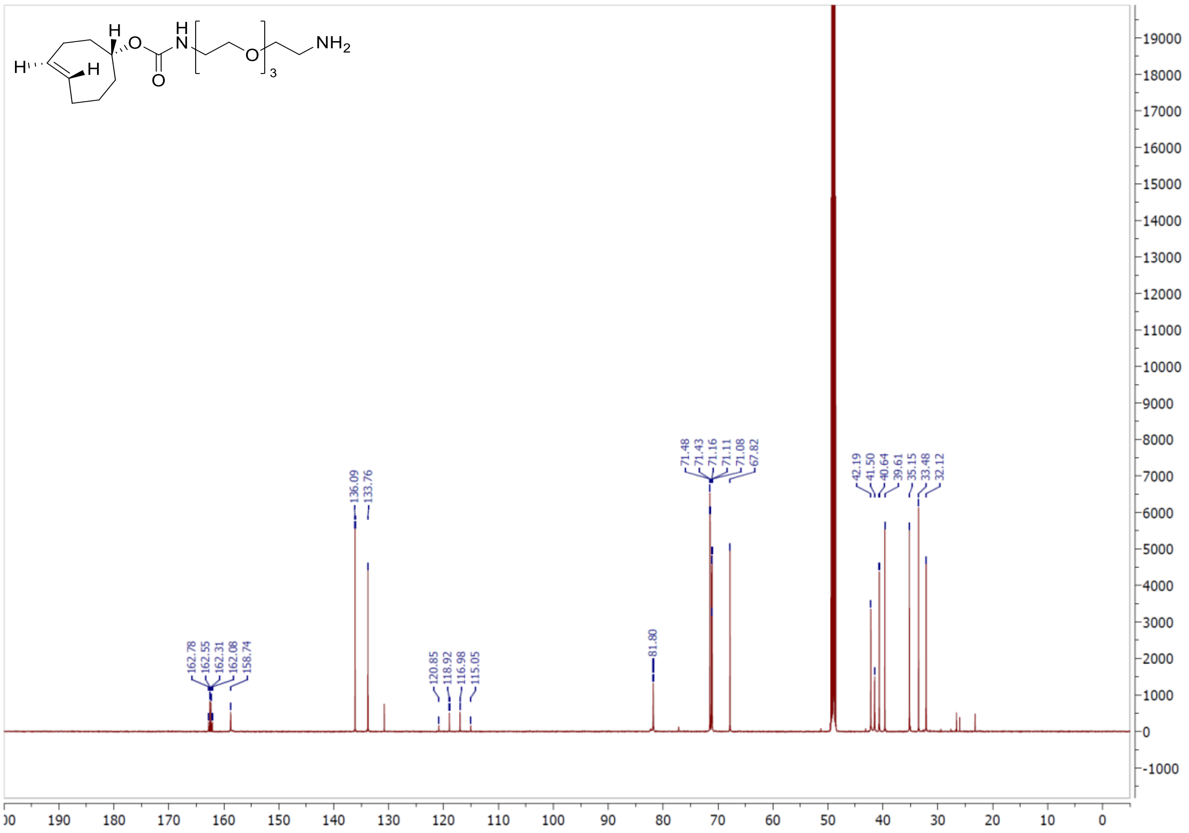

DOTA-TCO (7)

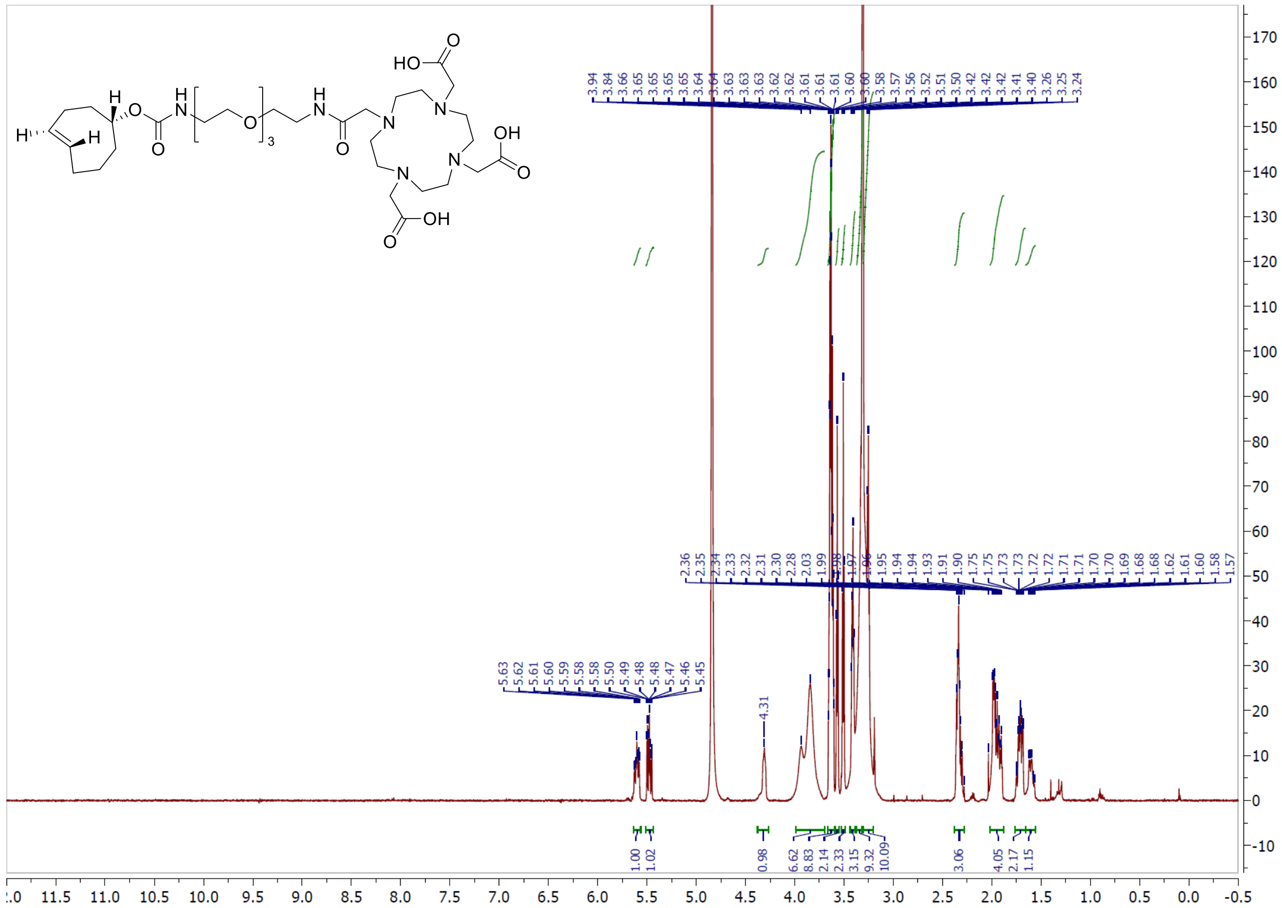

Supplement: Supplementary file 1 — Supporting Information [file CHEM-28-0-s001.pdf]
